# Nano‐Strainer: A workflow for the identification of single‐copy nuclear loci for plant systematic studies, using target capture kits and Oxford Nanopore long reads

**DOI:** 10.1002/ece3.10190

**Published:** 2023-07-18

**Authors:** Agnes Scheunert, Ulrich Lautenschlager, Tankred Ott, Christoph Oberprieler

**Affiliations:** ^1^ Evolutionary and Systematic Botany Group, Institute of Plant Sciences University of Regensburg Regensburg Germany

**Keywords:** CompCOS loci, *Leucanthemum*, polyploidization, probe kit, reticulation

## Abstract

In modern plant systematics, target enrichment enables simultaneous analysis of hundreds of genes. However, when dealing with reticulate or polyploidization histories, few markers may suffice, but often are required to be single‐copy, a condition that is not necessarily met with commercial capture kits. Also, large genome sizes can render target capture ineffective, so that amplicon sequencing would be preferable; however, knowledge about suitable loci is often missing. Here, we present a comprehensive workflow for the identification of putative single‐copy nuclear markers in a genus of interest, by mining a small dataset from target capture using a few representative taxa. The proposed pipeline assesses sequence variability contained in the data from targeted loci and assigns reads to their respective genes, via a combined BLAST/clustering procedure. Cluster consensus sequences are then examined based on four pre‐defined criteria presumably indicative for absence of paralogy. This is done by calculating four specialized indices; loci are ranked according to their performance in these indices, and top‐scoring loci are considered putatively single‐ or low copy. The approach can be applied to any probe set. As it relies on long reads, the present contribution also provides template workflows for processing Nanopore‐based target capture data. Obtained markers are further tested and then entered into amplicon sequencing. For the detection of possibly remaining paralogy in these data, which might occur in groups with rampant paralogy, we also employ the long‐read assembly tool canu. In diploid representatives of the young Compositae genus *Leucanthemum*, characterized by high levels of polyploidy, our approach resulted in successful amplification of 13 loci. Modifications to remove traces of paralogy were made in seven of these. A species tree from the markers correctly reproduced main relationships in the genus, however, at low resolution. The presented workflow has the potential to valuably support phylogenetic research, for example in polyploid plant groups.

## Graphical abstract

A workflow is presented which enables the identification of single‐/low‐copy nuclear molecular markers for a plant group of interest, by mining data from a small representative target capture experiment done using a commercial probe kit. Identified loci can be used for NGS amplicon sequencing and are particularly useful for researchers dealing with reticulate or polyploidization phylogenetic histories in plants.

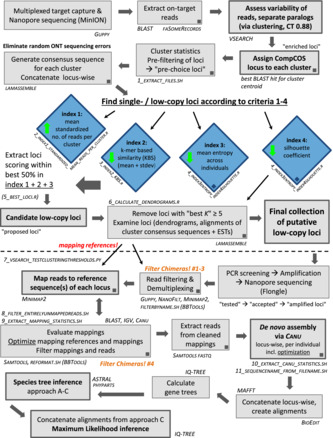

## 1 INTRODUCTION

In recent years, Next‐Generation Sequencing (NGS; Levy & Myers, 2016) has progressively enabled the analysis of complete plant or animal genomes for systematic research; to date, 2661 land plant and 10,670 metazoan genomic assemblies of varying quality are available in the Genome database at NCBI (NCBI Genome, 2023). However, conflicting signals among individual gene histories can distort the results of a joint analysis of a large number of genes. This is particularly prevalent in young lineages and those affected by polyploidization or reticulation, where hybridization or effects like incomplete lineage sorting (ILS) are prevalent (de Sousa et al., 2016; Knowles et al., 2018). Coalescent‐based approaches aiming at species tree reconstruction or constructing phylogenetic networks successfully deal with the problem but tend to be computationally intensive, which, in certain cases, may limit the number of loci that can be included in the analysis.

Genome reduction methods are suitable alternatives to avoid this issue, for example, high‐throughput amplicon sequencing (Brouwer et al., 2018). Another powerful approach is target enrichment via in‐solution hybridization, based on probes targeting a collection of loci. Specialized probe sets exist for various organism lineages and on various scales, from clade‐specific ones (Mascher et al., 2013; Villaverde et al., 2018) to those universally applicable in large groups like, for example, all Angiosperms (“Angiosperms353”, Johnson et al., 2019). However, target capture with commercial kits can be quite cost‐intensive, especially as in lineages featuring larger genomes, the efficiency of capture probes can become unfeasibly low. Jones et al. (2019), for example, reported the proportion of on‐target reads to be only 1.92% for the sample with the highest genome size (16.25 1C pg, i.e., approximately 15.9 Gbp in the haploid phase) included in their study.

Sequencing such large collections of loci sometimes even may be unnecessary, for example when aiming to reconstruct phylogenetic histories of groups prone to hybrid speciation or polyploidization. Despite ongoing optimization, for many tools intended for the analysis of allopolyploid origins, reticulation events in general, species delimitation, or species tree inference (e.g., allcopol, Lautenschlager et al., 2020; BPP, Flouri et al., 2018), scalability towards high numbers of markers can still be an issue. In studies focusing on reticulation or polyploidization histories (Oxelman et al., 2017; Rothfels, 2021), the absence of paralogy in the nuclear genes under investigation is much more important if specialized inference tools are to be used. Paralogs are pairs or groups of genes that started diverging by duplication instead of speciation (Fitch, 1970), for example, after a polyploidization event. Allopolyploidy on the other hand involves hybridization and results in pairs of genes called “homoeologous” per definition (Glover et al., 2016). Identifying parental homoeologs after an allopolyploidization event thus requires single‐copy markers to preclude the confounding effects of paralogy.

While commercial probe kits for target enrichment intend to target low‐ or single‐copy loci, uncertainty regarding the presence of paralogs increases with the taxonomic distance of a given study group relative to the lineage the probes were originally designed on, even when bait sets are regarded as “universal”. As long as no published genome of the study group is available, establishing single‐copy loci sets often is laborious and costly (but see Eserman et al., 2021).

In situations where (1) a smaller number of loci is sufficient for analysis, (2) single‐copy gene information is preferred and (3) no reference genome is available while (4) financial resources may be limited, an elegant solution would be to combine reduced target enrichment efforts with a low‐cost amplicon sequencing approach. We here introduce a bioinformatic pipeline for mining single‐ or low‐copy loci from a small‐scale target capture experiment (using only few representative taxa), based on inherent characteristics of the target capture data pointing towards non‐paralogy. The result is a phylogenetic marker set usable for elucidating complex phylogenetic relationships, for example, to disentangle hybrid speciation and polyploidization events; amplicons can be entered into NGS sequencing. Our pipeline is based on long‐read sequencing via Oxford Nanopore Technologies (Oxford, UK; ONT), which can be independently performed even in small labs at comparatively low cost. For groups where paralogy is particularly widespread, additional workflows are provided to enable identification and removal of traces of duplicated genes from the amplicon sequencing data. For this task, the Nanopore read assembly tool canu (Koren et al., 2017) is employed as well as a tree‐based orthology inference method, adapted from procedures introduced by Yang & Smith (2014).

We test our approach in the genus *Leucanthemum* Mill., a member of tribe Anthemideae in Asteraceae (Compositae). The family is characterized by rampant whole‐genome duplication (WGD) and a pronounced history of polyploidization (Barker et al., 2016; Huang et al., 2016; Watson et al., 2020). In addition, for the hyperdiverse daisy tribes (also named the “Fab Five”), which Anthemideae is a part of, reticulation due to ancient hybridization has been inferred (Watson et al., 2020), which further complicates phylogenomic inferences. The genus *Leucanthemum* itself is a large polyploidy complex comprising 42 species with ploidy levels ranging from diploid (2*x*) to dodecaploid (12*x*); *L. lacustre* (Brot.) Samp. even has 2*n* = 22*x* = 198 chromosomes (Euro+Med, 2023). The average genome size of the diploid species is approx. 11.7 Gbp in the diploid phase (based on entries of *L. vulgare*, *L. gaudinii*, *L. tridactylites*, and *L. laciniatum* in the Plant DNA C‐values Database, Leitch et al., 2019). A phylogenetic analysis of diploid *Leucanthemum* representatives, using amplified fragment‐length polymorphism (AFLP) fingerprinting data and a multilocus species tree reconstruction (based on nine low‐copy nuclear markers and the concatenated sequence information from five plastid intergenic spacer regions; Konowalik et al., 2015), corroborated earlier patterns of relationships found by examining the External Transcribed Spacer (ETS) region of the nuclear ribosomal repeat (Oberprieler et al., 2014): the species could be classified into an ancient, paraphyletic group and a second, monophyletic group of species. A subsequent Bayesian species tree reconstruction with *beast (Heled & Drummond, 2010) based on the same ten‐locus marker set (Wagner et al., 2019) confirmed the already known bipartition of *Leucanthemum* diploids into a well‐supported monophyletic group around *L. eliasii*, *L. legraeanum*, *L. ligusticum*, *L. monspeliense*, *L. pluriflorum*, and *L. vulgare*, and an unresolved, more ancient grade of the remaining diploids. This phylogenetic bipartition pattern was again found in a RADseq‐based study (using Restriction site‐associated DNA markers) by Ott et al. (2022), which mainly aimed at an integrative taxonomic treatment at the diploid level.

While for some of the polyploid species, their evolutionary relationships with diploid progenitors have been reconstructed (Greiner et al., 2012, 2013; Oberprieler et al., 2011, 2014), this has not yet been accomplished for the vast majority. Major obstacles have been the lack of a corroborated taxon delimitation based on established phylogenetic relationships at the diploid, “bottom” layer (but see Ott et al., 2022), but also the lack of a suitable molecular marker system for disentangling auto‐ and allopolyploid relationships in this young genus (crown age of diploids ca. 1–3 Ma; Wagner et al., 2019). This study was designed to lay the foundation to tackle these issues. Its aim is to (1) present a pipeline developed for mining single‐ or low‐copy nuclear markers from a small representative target capture dataset for subsequent long‐read amplicon sequencing, (2) test the pipeline on a young, polyploid genus with a documented history of polyploidization and large genome sizes, (3) evaluate whether markers suitable for species tree reconstruction can be obtained using our approach, (4) test whether the selected markers show any signs of paralogy, and if so, (5) determine how these paralogous remnants can be removed.

## 2 MATERIALS AND METHODS

**FIGURE 1:**
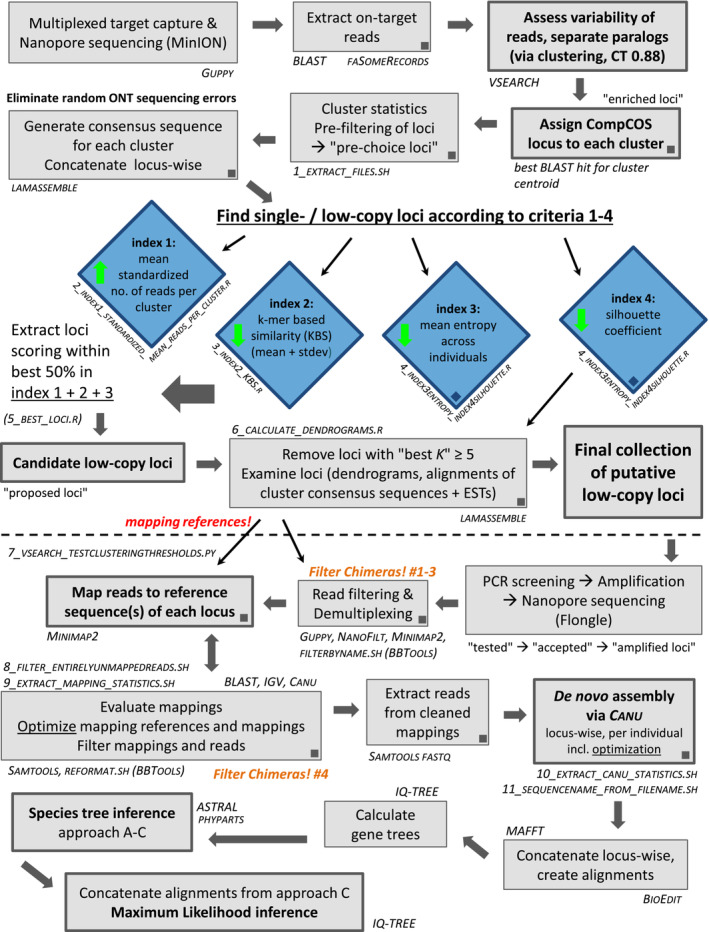
Illustration of the workflow presented in this paper, for analysis of target capture data, extraction of putative single‐/low‐copy loci, analysis of amplicon sequencing data from 13 + 2 amplified loci, and phylogenetic analyses. Tools or custom scripts necessary for each step are given beside boxes, squares within boxes denote steps where BASH commands were used (available in file “4_workflow_commands.docx” at Dryad). Central steps of the pipeline are the clustering and the assignment of reads to their respective CompCOS loci, and the selection of suitable loci based on criteria 1–4 via the four indices depicted by blue diamonds (green arrows inside the diamonds signify whether high or low values are “good” in the respective index). Important steps during the second part (below the dashed line) are the mapping of NGS reads from amplified loci to references, which are based on cluster consensus sequences, and the de novo assembly of reads using canu. Both steps include optimization procedures, which are described in detail in files 1–3 as available at Dryad. Steps intended to filter possible concatemers/chimeric sequences from the amplicon data are highlighted in orange. Finalized locus‐wise alignments are used for calculating gene and species trees.

For an easier overview of the numerous methods used in this study, Figure 1 graphically represents the workflow. Readers interested in applying the workflow to their own study group are advised to download two more detailed workflow figures alongside the detailed description of all wet‐lab and data analysis methods from Dryad (see Data Availability Statement), where also more information is given for each paragraph and all auxiliary software tools are mentioned.

### 2.1 Plant material and DNA extraction

Leaves of 43 individuals (accessions) from 20 *Leucanthemum* species were collected from 2004 to 2021 during field trips in France, Spain, Switzerland, Austria, Germany, Italy, Slovenia, Poland, Bosnia and Herzegovina, and Romania. The sampling thus includes all diploid species of the genus as accepted to date. Additionally, two outgroup samples from *Rhodanthemum catanache* and *Chlamydophora tridentata* were obtained from trips to Morocco and Cyprus, respectively. During the upstream target enrichment experiment, DNA from four *Leucanthemum* species was captured, each represented by a single accession; one of these accessions was also used for subsequent amplicon sequencing, the other three were replaced by other accessions. Voucher information and further details for all accessions are given in Table 1. Total genomic DNA was extracted using the CTAB protocol (Doyle & Dickson, 1987; Doyle & Doyle, 1987), in most cases from silica gel‐dried leaves (see Table 1).

**TABLE 1:** Taxa analyzed for the present study, alongside voucher information with localities, collectors and used Oxford Nanopore Technologies (ONT) barcodes.

Taxon	Sample ID	Voucher specimen(s)	Collection locality	Coordinates (latitude, longitude)	Collector, no.	Collection date	Library, run	Barcode
*Chlamydophora tridentata* (Del.) Less. (herbarium material)	A49	B100550374	CY, Larnaka, Meneou, 1 m	34.85, 33.61	*Vogt 8120*	13‐04‐1991	2 run1	BC21
*Rhodanthemum catananche* (Ball) B.H.Wilcox, K.Bremer & Humphries	R20‐01	B100704771	MA, Middle Atlas, Ifrane, Djebel Ari Benij, 2369 m	33.2554, −4.9663	*Vogt 17696 & al*.	12‐06‐2017	2 run1	BC20
*Leucanthemum ageratifolium* Pau	135‐07	B100386712	FR, Occitania, Pyrénées‐Orientales, 410 m	42.5038, 2.9603	*Konowalik KK42 & Ogrodowczyk*	05‐07‐2010	1 run1 + 2	BC01
*L. ageratifolium* Pau	M60‐01	B100345012, B100345013	ES, Castile‐La Mancha, Cuenca, 1157 m	40.1019, −1.521	*M. Cordel 60*	24‐06‐2009	1 run1 + 2	BC02
*L. burnatii* Briq. & Cavill.	90‐01	B100464678	FR, Provence‐Alpes‐Côte d'Azur, Alpes‐Maritimes, 1235 m	43.7607, 6.9165	*Vogt 16615 & al*.	27‐05‐2010	1 run1 + 2	BC03
*L. burnatii* Briq. & Cavill.	92‐02	B100464676, B100464675	FR, Provence‐Alpes‐Côte d'Azur, Bouches‐du‐Rhône, 650 m	43.545, 5.6626	*Vogt 16618 & al*.	29‐05‐2010	1 run1 + 2	BC04
*L. cacuminis* Vogt, Konowalik & Oberpr.	62‐02	B100413744	ES, Galicia, 750 m	42.9249, −6.8657	*Hößl 62*	06‐08‐2007	1 run1 + 2	BC05
*L. cacuminis* Vogt, Konowalik & Oberpr.	68‐01	B100413738	ES, Cantabria, 1770 m	43.1538, −4.8053	*Hößl 68 & Himmelreich*	09‐08‐2007	1 run1 + 2	BC06
*L. eliasii* (Sennen & Pau) Vogt, Konowalik & Oberpr.	504‐01	B101189931	ES, Castile and León, Burgos, 890 m	42.5083, −3.7011	*Vogt 18077 & Prem‐Vogt*	19‐06‐2021	2 run1	BC18
*L. eliasii* (Sennen & Pau) Vogt, Konowalik & Oberpr.	510‐01	B101189938	ES, Castile and León, Burgos, 995 m	42.69, −3.2656	*Vogt 18085 & Prem‐Vogt*	20‐06‐2021	2 run1	BC19
*L. gallaecicum* Rodr.Oubiña & S.Ortiz	159‐11	B100386789, B100420775, B100464989	ES, Galicia, Pontevedra, 375 m	42.8498, −7.9878	*Konowalik KK67 & Ogrodowczyk*	08‐07‐2010	1 run1 + 2	BC07
*L. gallaecicum* Rodr.Oubiña & S.Ortiz	161‐06	No voucher	ES, Galicia, Corunna, 380 m	42.8533, −7.9994	*Konowalik s.n. & al*.	09‐08‐2010	1 run1 + 2	BC08
*L. gaudinii* Dalla Torre	209‐01	B100386664	CH, Bern, Interlaken‐OberhaslIT, 2262 m	46.5781, 7.97	*Tomasello TS88*	09‐08‐2010	1 run1 + 2	BC09
*L. gaudinii* Dalla Torre	276‐01	B100413015	AT, Carinthia, Feldkirchen, 2270 m	46.8603, 13.8172	*Oberprieler 10866*	18‐09‐2011	1 run1 + 2, target	BC10, BC05*
*L. gracilicaule* (Dufour) Pau	84‐10	B100386704	ES, Valencian Community, Alicante, 296 m	38.8379, −0.1853	*Konowalik KK20 & Ogrodowczyk*	12‐05‐2010	1 run1 + 2	BC11
*L. gracilicaule* (Dufour) Pau	85‐04	B100386702	ES, Valencian Community, Valencia, 337 m	39.3135, −0.681	*Konowalik KK25 & Ogrodowczyk*	14‐05‐2010	1 run1 + 2	BC12
*L. graminifolium* (L.) Lam.	116‐01	B100464684, B100464683	FR, Occitania, Hérault, 802 m	43.7761, 3.2386	*Vogt 16693 & al*.	31‐05‐2010	1 run1 + 2	BC13
*L. graminifolium* (L.) Lam.	96‐06	B100464663	FR, Occitania, Aude, 600 m	43.1494, 2.6294	*Vogt 16656 & al*.	30‐05‐2010	1 run1 + 2	BC14
*L. halleri* (Vitman) Ducommun	162‐03	B100386798	DE, Bavaria, Garmisch‐Partenkirchen, 2345 m	47.4134, 11.1277	*Konowalik KK67 & Tomasello*	02‐10‐2010	1 run1 + 2	BC15
*L. halleri* (Vitman) Ducommun	208‐01	B100386672	CH, Valais, Sitten, 2320 m	46.3308, 7.2911	*Tomasello TS65*	04‐08‐2010	1 run1 + 2	BC16
*L. laciniatum* Huter & al.	280‐03	B100464203	IT, Calabria, Cosenza, 1580 m	39.902, 16.1144	*Tomasello TS420*	25‐08‐2011	1 run1 + 2	BC17
*L. laciniatum* Huter & al.	280‐05	B100464203	IT, Calabria, Cosenza, 1580 m	39.902, 16.1144	*Tomasello TS420*	25‐08‐2011	1 run1 + 2	BC18
*L. legraeanum* (Rouy) B.Bock & J.‐M.Tison	366‐01	B100486634, B100486635, B100486636, B100486637, B100486638	FR, Provence‐Alpes‐Cote d'Azur, Var, 410 m	43.1986, 6.3151	*Vogt 17189*	18‐06‐2013	1 run1 + 2	BC19
*L. legraeanum* (Rouy) B.Bock & J.‐M.Tison	369‐01	B100486648, B100486649	FR, Provence‐Alpes‐Cote d'Azur, Var, 210 m	43.2444, 6.3377	*Vogt 17192*	18‐06‐2013	1 run1 + 2	BC20
*L. ligusticum* Marchetti, R.Bernardello, Melai & Peruzzi	406‐01	B100627838, B100627839	IT, Liguria, La Spezia, 210 m	44.247, 9.7728	*Vogt 17460 & al*.	01‐06‐2015	1 run1 + 2	BC21
*L. ligusticum* Marchetti, R.Bernardello, Melai & Peruzzi	412‐01	B100627849, B100627850, B100627851	IT, Liguria, Genova, 700 m	44.3603, 9.5105	*Vogt 17467 & al*.	02‐06‐2015	2 run1	BC01
*L. lithopolitanicum* (E. Mayer) Polatschek	273‐02	B100413012	SL, Central Slovenia, 2100 m	46.3633, 14.5715	*Oberprieler 10862*	17‐09‐2011	2 run1	BC02
*L. lithopolitanicum* (E. Mayer) Polatschek	274‐01	B100413013	SL, Savinja, 1999 m	46.375, 14.5663	*Oberprieler 10864*	18‐09‐2011	2 run1	BC03
*L. monspeliense* (L.) H.J.Coste	128‐01	B100464618	FR, Occitania, Gard, 750 m	44.0888, 3.5786	*Vogt 16712 & al*.	01‐06‐2010	2 run1	BC04
*L. monspeliense* (L.) H.J.Coste	340‐01	B100486666, B100486667	FR, Occitania, Aveyron, 184 m	44.5822, 2.184	*Vogt 17156 & al*.	04‐06‐2013	2 run1	BC05
*L. monspeliense* (L.) H.J.Coste	131‐01	B100464615	FR, Occitania, Gard, 380 m	44.1412, 3.7316	*Vogt 16716 & al*.	01‐06‐2010	targent	BC04*
*L. pluriflorum Pau*	55‐03	B100413749	ES, Galicia, Lugo, 10 m	43.6309, −7.333	*Hößl 55*	02‐08‐2007	2 run1	BC06
*L. pluriflorum Pau*	40‐09	B100413758	ES, Galicia, Corunna, 100 m	42.8838, −9.2726	*Hößl 40*	24‐07‐2007	2 run1	BC07
*L. pyrenaicum* Vogt, Konowalik & Oberpr.	267‐03	B100464210	ES, Aragon, Huesca, 2000 m	42.6327, 0.453	*Tomasello TS392*	29‐07‐2011	2 run1	BC08
*L. pyrenaicum* Vogt, Konowalik & Oberpr.	266‐02	B100464208	ES, Aragon, Huesca, 1650 m	42.7806, −0.2467	*Tomasello TS382*	27‐07‐2011	2 run1	BC09
*L. rotundifolium* (Willd.) DC.	446‐01	No voucher	PL, Podkarpackie, Bieszczady, 920 m	49.1191, 22.5776	*Konowalik 20180622‐02‐01*	22‐06‐2018	2 run1	BC10
*L. rotundifolium* (Willd.) DC.	449‐01	No voucher	BH, Dinaric Alps, Vranica Planina	43.9578, 17.7403	*Konowalik 20180807‐03‐01*	07‐08‐2018	2 run1	BC11
*L. rotundifolium* (Willd.) DC.	495‐02	B101068540, B101068541	RO, 1440 m	45.404, 22.8857	*Wagner FW‐01 & Maier*	18‐08‐2019	Targent	BC06*
*L. tridactylites* (A.Kern. & Huter) Huter & al.	278‐01	B100464207	IT, Abruzzo, Pescara, 2080 m	42.1384, 14.1101	*Tomasello TS417*	24‐08‐2011	2 run1	BC12
*L. tridactylites* (A.Kern. & Huter) Huter & al.	278‐05	B100464207	IT, Abruzzo, Pescara, 2080 m	42.1384, 14.1101	*Tomasello TS417*	24‐08‐2011	2 run1	BC13
*L. virgatum* (Desr.) Clos	225‐01	B100411746	FR, Provence‐Alpes‐Côte d'Azur, Alpes‐Maritimes, 430 m	43.9538, 7.2961	*Vogt 16892 & Oberprieler 10802*	11‐06‐2011	2 run1	BC14
*L. virgatum* (Desr.) Clos	250‐01	B100350169, B100350172	IT, Liguria, Savona, 215 m	44.0596, 8.0583	*Vogt 16932 & Oberprieler 10839*	13‐06‐2011	2 run1	BC15
*L. vulgare* Lam.	184‐01	B100346626	BH, Republic of Srpska, Nevesinje, 930 m	43.2403, 18.3364	*Vogt 16806 & Prem‐Vogt*	26‐07‐2010	2 run1	BC16
*L. vulgare* Lam. (liquid nitrogen)	L046‐08	B100550249	DE, Bavaria, Regensburg, 450 m	49.0333, 11.8833	*C. Eder & C. Oberprieler*	08‐06‐2004	2 run1	BC17
*L. vulgare* Lam.	120‐02	B100464627	FR, Occitania, Aveyron, 756 m	43.8925, 3.2477	*Vogt 16699 & al*.	01‐06‐2010	targent	BC02*

*Note*: Sample IDs identify the population and the respective individual sampled. All vouchers were deposited at the herbarium of the Botanical Garden, Freie Universität Berlin (B). Unless stated otherwise, all accessions were conserved in silica gel. For each sample, the library (1 or 2) and the number of sequencing runs (two or one) are given. Used barcodes pertain to the ONT Native Barcoding Expansions 1–12 and 13–24 (EXP‐NBD104/EXP‐NBD114). Four samples were used in the target enrichment (“targent”) experiment; the respective barcodes are marked with an asterisk (*) and are from the ONT PCR Barcoding Expansion 1–12 (EXP‐PBC001).

Abbreviations: AT, Austria; BH, Bosnia and Herzegovina; CH, Switzerland; CY, Cyprus; DE, Germany; ES, Spain; FR, France; IT, Italy; MA, Morocco; PL, Poland; RO, Romania; SL, Slovenia.

### 2.2 Target enrichment in four representative species

The target enrichment experiment was performed using the Arbor Biosciences myBaits capture kit “COS Compositae/Asteraceae 1Kv1”/“Compositae‐1061”, developed for the Asteraceae by Mandel et al. (2014). The “CompCOS loci” (COS: Conserved Ortholog Set) are based on alignments of *Helianthus*, *Lactuca*, and *Carthamus* Expressed Sequence Tags (ESTs) against *Arabidopsis* spliced gene models; the probe kit, consisting of 9678 baits, targets 1061 nuclear loci, which are conserved within Asteraceae, with several of them supposed to be single‐ or low‐copy. The kit has been used successfully on various taxonomic scales, from species complexes across single genera to tribal relationships up to the Asteraceae tree of life (Jones et al., 2019; Mandel et al., 2017, 2019; Watson et al., 2020). Captured DNA fragments were sequenced on an ONT MinION Mk1B device. As ONT does not provide an official protocol for multiplexed capture of more than one sample, we here present a new combined workflow comprising elements from the Nanopore “Sequence capture” protocol, the Nanopore “PCR barcoding genomic DNA” protocol, and the official myBaits manual.

Sheared and size‐selected DNA (targeted fragment length: 1500–4000 bp) was barcoded for each sample, using the PCR Barcoding Expansion 1–12 (EXP‐PBC001, see Table 1). The target enrichment was performed following the myBaits manual except for some modifications. As neither Arbor Biosciences nor ONT offered blockers for Nanopore libraries, we individually designed six blocking oligos, four of them also usable for post‐capture multiplex amplification and consisting of the forward sequence of the used Nanopore barcodes alongside their 5′ flanking region (Table 2, “*BC0x_prime_block*”), and two corresponding to the forward sequences of the two Y‐arms of the barcode adapter BCA from the PCR Barcoding Expansion (“*BCA_block_1st/2nd*”). Hybridization was performed at 65°C for 24 h (for a step‐by‐step account on the procedure refer to the “detailed methods” document at Dryad). Library prep was finished according to the Nanopore Sequence capture protocol using the Nanopore Ligation Sequencing kit (SQK‐LSK109), and the library sequenced on a Nanopore MinION Mk1B using a FLO‐MIN106 flow cell.

**TABLE 2:** Oligonucleotides used for blocking barcode and adapter sequences during the in‐solution hybridization.

Oligo name	Sequence 5′–3′	Length
BC02_prime_block	GTTCGTAA GGTGCTG TCGATTCCGTTTGTAGTCGTCTGT	39 bp
BC04_prime_block	GTTCCTAC GGTGCTG TTCGGATTCTATCGTGTTTCCCTA	39 bp
BC05_prime_block	ATTCATAC GGTGCTG CTTGTCCAGGGTTTGTGTAACCTT	39 bp
BC06_prime_block	GTTCCTAG GGTGCTG TTCTCGCAAAGGCAGAAAGTAGTC	39 bp
BCA_block_1st	TTTCTGTTGGTGCTGATATTGC‐p	22 bp
BCA_block_2nd	TACTTGCCTGTCGCTCTATCTTC‐p	23 bp

*Note*: Barcode blockers (*BC0x_prime_block*) include the 5′ flanking region of the Nanopore barcodes, plus 8 random basepairs at the 5′ end (the 3′ flanking region of the barcodes remained unblocked). The barcode blockers were also used for post‐capture amplification of the library; the blockers *BCA_block_1st* and *BCA_block_2nd* (blocking the sequences of the two Y‐arms of the Nanopore Barcode Adapter BCA) were prevented from acting as primers by being phosphorylated at their 3′ ends.

### 2.3 Analysis of target capture data and choice of suitable loci for amplicon sequencing

In summary, assignment of reads to probe genes as well as assessment of intra‐locus genetic variability and paralog separation is done by a sequential clustering approach that eventually leads to a certain number of representative sequences (i.e., cluster consensus sequences) for each locus and species. These sequences are then examined for properties regarded indicative of single‐ or low‐copy loci (see Chapter 2.3.4). Many of the analyses and necessary data handling steps described have been carried out using BASH and/or AWK commands on a Linux workstation. These commands alongside explanations are provided at Dryad, together with parameters as applied for tools and software; the latter can also be found in the “detailed methods” document. Where specialized scripts were used, they are mentioned here; all of them can likewise be downloaded from Dryad as well as GitHub, together with an explanatory text file.

#### 2.3.1 Extraction of on‐target reads

To determine on‐target reads, the FASTA read files were locally BLASTed, using blast+ v.2.12.0 (Altschul et al., 1990; Camacho et al., 2009), against the collection of source ESTs originally used to design the CompCOS capture probes (Mandel et al., 2014; hence referred to as “CompCOS ESTs”). From the collection of all reads for each individual, those with a BLAST hit were then extracted with the fasomerecords application (Kent Utilities, n.d., download website see References).

#### 2.3.2 Clustering and assignment of reads to their respective CompCOS locus

The obtained on‐target reads were subjected to a clustering process, separating reads divergent beyond a certain threshold, followed by the assignment of each cluster to its respective CompCOS locus. The clustering step was performed using vsearch v.2.15.0 (Rognes et al., 2016), including appropriate adjustment of the clustering threshold (CT) parameter. Among others, vsearch produced FASTA files with centroids and cluster consensus sequences of all clusters. The partially overlapping raw reads within each cluster were then aligned using the local version of lamassemble (Frith et al., 2021; see also Katoh et al., 2019). After alignment, all cluster files were renamed according to the best CompCOS BLAST hit of their centroids (for details refer to the “detailed methods” document as well as file “4_workflow_commands.docx” at Dryad). As most ESTs received multiple clusters, the latter were numbered sequentially for each EST.

#### 2.3.3 Loci summary statistics and pre‐choice loci

In order to enable a thorough examination of the loci, and also for calculation of the first of the four indices (see below), loci summary statistics on the clustering of each sample and across all individuals were calculated first, among others the numbers of clusters with less than five reads (here referred to as “low‐coverage clusters”).

With the above‐mentioned statistics at hand, obviously unqualified loci were excluded in a first step to ease processing. For example, loci with a very high number of reads are unlikely to be single‐copy, while a minimum of reads is needed for calculation of indices, and generally to reliably reconstruct marker sequences from Nanopore raw reads with relatively high error rates. Consequently, as a first approximation, loci with fewer than 100 and more than 1000 sequenced reads were excluded. Furthermore, only loci with reads present in three or more individuals and with at least one cluster (per individual) containing five or more reads were kept. Clusters from these “pre‐choice loci” were extracted for each individual (script 1_extract_files.sh) from its respective folder containing all aligned, renamed cluster files (result of 2.3.2); singleton clusters (i.e., clusters with only one read) and low‐coverage clusters were dismissed (see Section 4). For the remaining clusters, their consensus sequences were calculated from the alignments via lamassemble, and the consensus sequences of all individuals combined locus‐wise into common FASTA files. These FASTA files served as input for the scripts calculating indices 2–4 (see below).

#### 2.3.4 Choice of putatively non‐paralogous loci, based on four specialized indices

The crucial step in the analysis of the target enrichment data was the selection of the best loci given the *Leucanthemum* data and, more generally, deciding on what are appropriate criteria for defining a “good” (i.e., single‐ or low‐copy) locus. Given a collection of reads, it is not possible to infer single‐ or low‐copy loci directly. For instance, the number of reads in each locus might not only be influenced by the amount of copies, but also by the amount of data sequenced, sequence properties, biased amplification, or degree of identity with the used probe. The number of reads per locus can thus only be used for very rough filtering of eligible loci (see above). Instead, indirect criteria have to be used. Here, we assumed a locus likely to be single‐ or low‐copy if it met the four following criteria:

1. It has many reads per cluster; given a certain number of reads, few clusters in a locus can be (among other factors, e.g., sequence characteristics) a sign of low locus divergence; a low total number of clusters per se is not indicative, as few clusters could also be caused by a low total number of reads.
2. Ideally, all cluster consensuses from the same species are more similar to each other, than to clusters from another species (indicating the absence of paralogy that is sufficiently ancient for detection).
3. When calculating distance‐based dendrograms from cluster consensus sequences, consensuses belonging to the same species cluster together, instead of being scattered all over the tree (similar to criterion (2) but representing a tree‐based measure).
4. When calculating distance‐based dendrograms from cluster consensus sequences, only few main clades/groups are found (indicating no to few paralogous groups).

These four criteria were assessed by calculating four specialized metrics (here called indices) for each of the 467 pre‐choice loci. We provide three custom R scripts to accomplish this task.

The first index, corresponding to criterion (1), is the mean standardized number of reads per VSEARCH cluster in a locus. The index is based on the complete cluster collection. It was calculated based on the loci summary statistics of the pre‐choice loci: for each individual *i* and locus *L*, the number of reads from *i* assigned to *L* is divided by the corresponding number of VSEARCH clusters found for *L* in *i*. For each individual, the resulting average numbers of reads per cluster are then standardized by calculating their *z*‐score. Finally, for each locus, these standardized values are averaged across individuals. The standardization step aims to prevent this ranking criterion from being dominated by single individuals. The higher the resulting value, the “better” the locus in terms of low sequence divergence within it. Calculations are performed by the R script 2_index1_standardized_mean_reads_per_cluster.r.

Regarding criterion (2), we calculated a “*k*‐mer‐based similarity (KBS)” index, defined as the average pairwise *k*‐mer distance between the several consensus sequences of ONE species, relative to the average pairwise *k*‐mer distance between consensus sequences of ALL species. The index is calculated per species and provides the percent deviation given a locus‐wide average distance between all consensuses. The underlying rationale is that in a single‐copy locus, cluster consensuses from the same species will ideally be more similar to each other than to consensuses from another species. In such a case, the KBS index will be negative. Positive values on the other hand represent a situation where sequences of different species are usually more similar than those from the same species. To obtain a locus‐wide quality measure, the mean KBS index of all four species can be used: in a “good” locus, where all species take on negative values, the overall mean KBS index will be low as well. The standard deviation of the species' mean KBS indices provides information on how much the species differ in terms of their KBS. A perfect locus should thus be characterized by a low mean KBS index and a low standard deviation. *K*‐mer distances (*k*‐mer length = 8) were used to compute this measure because consensus sequences might be too divergent to produce reliable multiple sequence alignments, which are a precondition for common distance measures such as the p‐distance. For each pair of consensuses, their *k*‐mer distance was calculated using the k mer package (Wilkinson, 2018) All calculations were implemented in the R script 3_index2_kbs.r.

**FIGURE 2:**
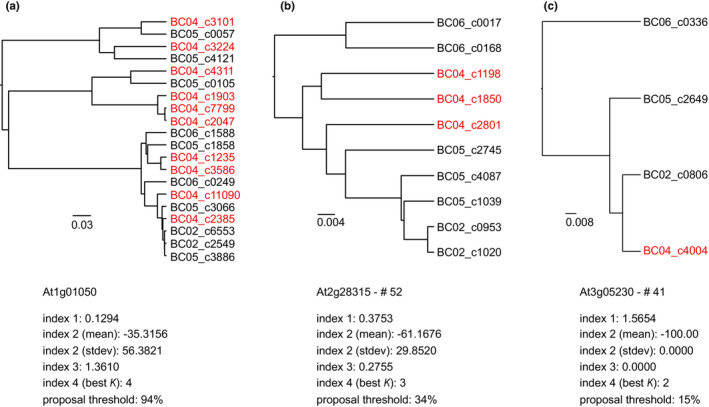
Exemplary dendrograms as used during computation of indices 3 and 4 and for visual inspection, from three CompCOS loci targeted in the target capture experiment (a: *At1g01050*, contained in the pre‐choice loci but not in the proposed loci; b: *At2g28315*, c: *At3g05230*, both contained in the amplified loci). Leaves are consensus sequences of VSEARCH clusters assigned to the respective locus, and dendrograms are based on single‐linkage hierarchical clustering using pairwise *k*‐mer distances. Leaf names contain the number of the respective cluster as well as the Nanopore barcode of the individual the reads belong to (BC02–BC06). In most cases, each individual is represented by several clusters (as highlighted in red for BC04, *L. monspeliense*). Corresponding index values of the respective markers are given below each dendrogram. index 1: mean standardized number of reads per cluster; index 2: *k*‐mer‐based similarity; index 3: mean entropy; index 4: silhouette coefficient (best *K*).

Computation of indices for criteria (3) and (4), as well as visual inspection of chosen candidate loci (see 2.3.5), required dendrograms to be computed for each locus. These were generated via single‐linkage hierarchical clustering of the aforementioned *k*‐mer distances between cluster consensus sequences in a given locus. Single‐linkage clustering is recommended here as it enables clustering of shorter consensuses (representing different fractions of an EST) together with longer ones that cover the whole EST and thus helps reduce the effect of artificial splitting. Computation of dendrograms was done using the ape (Paradis & Schliep, 2019) package in r via rstudio (R Core Team, 2021; RStudio Team, 2020) and is part of the script calculating indices 3 and 4 as well as of script 6_calculate_dendrograms.r. The dendrograms of three exemplary loci are shown in Figure 2.

Criterion (3) is based on similar theoretical considerations as criterion (2), but similarity here is evaluated using dendrograms instead of mere *k*‐mer distances. Here, the goal is to detect (and dismiss) loci where the sequences (leaves) of a given species are scattered across the locus' dendrogram instead of being grouped together. This is achieved by measuring the uncertainty to which clade an individual belongs by means of the Shannon entropy. However, it is required that the presumable number of main clades in a dendrogram is known. The script 4_index3entropy_index4silhouette.r provides this information (see below) and also performs entropy computation in the following way:

Let *I* be the number of individuals and *n*_*ik*_ denote the number of leaves (= consensus sequences of vsearch clusters) from individual *i*, which are assigned to clade *k*. Then, the entropy of the *i* ^th^ individual can be computed as $H_{i}=-\sum_{k=1}^{K}\left(p_{\mathrm{ik}}\log\;p_{\mathrm{ik}}\right)$, where $p_{\mathrm{ik}}=n_{\mathrm{ik}}/\sum_{k=1}^{K}\;n_{\mathrm{ik}}$ and $p_{\mathrm{ik}}\log\;p_{\mathrm{ik}}≔0$ if $p_{\mathrm{ik}}=0$. As a ranking criterion, the entropy was averaged across individuals using each individual's number of consensus sequences as weights:

$$\overset{‐}{H}=\frac{\sum_{i=1}^{I}H_{i}\sum_{k=1}^{K}n_{\mathrm{ik}}}{\sum_{i=1}^{I}\sum_{k=1}^{K}n_{\mathrm{ik}}}$$

In this index 3 again, low values argue against paralogy.

As mentioned above, criterion (4) was likewise assessed by script 4_index3entropy_index4silhouette.r. This was done by calculating the silhouette coefficients (Kaufman & Rousseeuw, 1990) for various cuts through a given dendrogram, and considering the best value of *K* (corresponding to the highest coefficient) as the presumable number of main clades (e.g., *K* = 3 in Figure 2b). A high optimal *K* may indicate the presence of high sequence divergence and likely multiple paralogs in a locus that, therefore, should be dismissed. As the script for ultimately selecting the candidate loci (see below) uses percentiles of values from continuous indices, the discrete silhouette index 4 could not be sensibly incorporated into the ranking for the “best” loci. Instead, we used it as an additional criterion to exclude possible paralogous loci from the collection of candidates.

In a final step, the top‐scoring loci were extracted using the script 5_best_loci.r, based on locus data for index 1, 2 (mean and standard deviation), and 3. We defined a “good” locus as one which performed well in ALL indices. The script ranks loci according to their performance in each index, by calculating their respective percentiles, and outputs those loci, which are among the best 1%, 2%, …, 99% of all loci (i.e., up to the respective percentile threshold) with respect to all index values. Naturally, in this way, the number of proposed loci increases with higher percentages/percentiles thresholds; this means that from the output, a suitable “proposal threshold” can be chosen depending on how many loci are needed. Candidate loci with a best *K* value of 5 or more in the silhouette index calculation (index 4) were excluded from the collection (in a tree containing four species, a maximum of four main clades might theoretically be explained by strong phylogenetic signal influencing the best *K*).

#### 2.3.5 Assessment of candidate loci

To evaluate the ability of the indices to predict single−/low‐copy loci in the dataset, and to further refine the choice of loci prior to wet‐lab screening, dendrograms were computed for visual inspection for all obtained candidate loci using the script 6_calculate_dendrograms.r. Furthermore, in each locus's FASTA file (with cluster consensus sequences of all individuals), the sequences were aligned alongside the respective source ESTs using again lamassemble. As substantial divergence occurred among consensus sequences of some markers, these alignments were unsuitable for further analysis and were only used for rough visual inspection and primer design, and, modified and processed further, for generation of references for mapping, see 2.4.4. Upon inspection of dendrograms and alignments, loci with strongly divergent consensus sequences within or among individuals, or conspicuous inter‐individual groupings leading to dendrograms comprising long basal branches or several clades with individuals' sequences scattered across the tree, were excluded from further processing.

### 2.4 Amplicon sequencing

#### 2.4.1 Primer design and PCR screening

All loci for which primers could be designed were tested in a PCR screening approach (for details on this procedure as well as others described in the entire Chapter 2.4. (Amplicon sequencing) refer to the “detailed methods” document at Dryad). Markers producing clear multiple bands in two or more individuals were regarded unsuitable and dismissed, as well as markers failing to amplify any product. All remaining markers can generally be regarded as acceptable, but for the purpose of the present study, markers for which primers failed to amplify clear bands in one or two individuals in the screening, or markers passing the PCR screening but having only low variability were excluded as well. Average values for indices 1, 2 (mean and standard deviation), and 3 were calculated for accepted versus non‐accepted markers separately and the results examined for differences. Trends were tested for significance for each of the index values separately using one‐sided Mann–Whitney *U* tests (coding see Table 3).

**TABLE 3:** Proposed candidate loci alongside additional information.

Locus (no.)	Forward primer (5′–3′)	Reverse primer (5′–3′)	Annotated gene in *Artemisia*	Aligned length	Estim. Variab.	Screen: Ind. Mult. Bands	Screen: Ind. Amplified	Status after PCR screening	Amplif.: Ind. Mult. Bands	No. seq. approach A	No. seq. approach B	No. seq. approach C/concat. ML
At1g04290 (#39)	CACAACAGCAACAGCCTTCC	TGGTGCGACTACAACAGGTG	Thioesterase superfamily protein	2043	Med	‐‐	3	Accepted	‐‐			
At2g21870 (#40)	AGTCCCTGTTCGCTGCTTTG	GCTCTTCGTATTTAGGCAAGTCC	MALE GAMETOPHYTE DEFECTIVE 1	3035	Low	‐‐	3	Accepted	‐‐			
At3g05230 (#41)	TCTGCCAACCTACAGTCACTG	AACTCTGGCAAGCGAAAACC	Signal peptidase 22 kDa subunit	889	Med	‐‐	4	Amplified	0	41	41	41
At4g36440 (#42)	TGCGAGAAATCGGGTCAACG	TGCCCGGAAGTGCATCAATC	Hypothetical protein	1554	Low	‐‐	4	Accepted	‐‐			
At5g65380 (#43)	GGTCCTGCCATCTTCAGTCG	AGACAAGGCCTATCACCGAAG	MATE efflux family protein	2492	Med	‐‐	2	Accepted	‐‐			
At1g68570 (#44)	GAGCTTGACACCTTTGCTTGG	AGCTCAACCTCATGATCTTTGTG	Proton‐dependent oligopeptide t. f.	1719	Med	1 (2)	4	Amplified	2?	41	41	41
At3g46630	No primer design possible	n.a.	n.a.	n.a.	n.a.	n.a.	n.a.	n.a.				
At5g17560 (#45)	GCTGCTTCTTCGGGAGTCTT	TCAATGAYGCTTATGGTGATGG	BoIA(−like) protein	2111	High	‐‐	4	Amplified	12*	53	34/17	15/17
At4g11560 (#46)	TCAACTCATTAGGTGACATGTTCAG	CAAACAAGAAGCCKTATGTAGC	Bromo adjacent homology (BAH) d.‐c. p.	1919	Med	‐‐	4	Amplified	0	28	28	28
At4g18593 (#47)	ACAGTGCTGATATCGACTCG	TGCGGCTGATCCTGCATTAG	Dual specificity phosphatase	913	Low	‐‐	4	Accepted	‐‐			
At5g14130 (#9A)	GTTGGTTCTCGCCGGTAGAG	TGATCTGATGTAAACAAGCCCTT	Peroxidase superfamily protein	1505	High	2 (2 faint)	4	Amplified	17*	40	40	38
At3g03100 (#49)	AGATTCACAATATAGGTGCCACAT	GGTCTTGGTGGGTTGCCAA	NADH:ubiquinone o.r., 17.2 kDa subunit	1412	Low	4 (3)	4	Not accepted	‐‐			
At1g61040 (#50)	ACGCAGATAGAAGAAGGCTTTC	GCCTCCTCCCATCATTCTCAG	Plus‐3	1483	Low	‐‐	4	Accepted*	0	42	42	30
At1g70160 (#51_1)	ACAGTTGGACATGTATGGATCTCT	CCCACCATTCATCCCAAGGA	Hypothetical protein	1979	High	‐‐	4	Amplified	5	44	41	32
(#51_2)	GGGGCATTTGCTGGTCATAC	GCAGTACTCGTTCATGTTGGC		1959	High	1 (2)	4	Amplified	Failed			
At2g28315 (#52)	GTCTTTGGTTGCTTTCTTCACTTC	AGCCTTGATGAGCAATCTTGG	Nucleotide/sugar transporter family p.	2051	Med	‐‐	4	Amplified	3?	36	36	36
At4g32140 (#53)	CTCTCTCGTTGGCGATCTGT	GAACCAAGAATATAAATGGCAGAA	eamA‐like transporter family	1277	Med	‐‐	3	Accepted	‐‐			
At1g79230 (#54)	AGTTCGTAGTGATATGGCTTCAA	CCTCTGTTCATCGGGCATGT	Tetratricopeptide‐like helical d.‐c. p.?	934	High	1 (2)	4	Amplified	7*	54	33/16	33/16
At3g50690 (#55)	GCGCCGTTAAGTGTGTTCAG	TCCTCCTCATCCACTTCAGGA	Leucine‐rich repeat‐containing protein	2822	Med	‐‐	***0***	Not accepted	‐‐			
At1g11790 (#56)	CCTTATCAGCGACGGATCTTG	TTCTGCAGCCCGAGAACTTG	Arogenate dehydratase	2470	Med	‐‐	4	Amplified	8–9	39	39	39
At5g62670 (#57)	CGAGTCCATTGCTGCTTTACC	TGACCACTGATTCAACATGACC	Plasma membrane ATPase	2519	Med	‐‐	4	Amplified	0	43	43	34
At1g53645	No primer design possible	n.a.	n.a.	n.a.	n.a.	n.a.	n.a.	n.a.				
At2g45030 (#13A)	TCACGCAGGTCAAATTGTAGC	TGGAACGAAGAGCTGTTGAG	Hypothetical protein	1710	High	‐‐	4	Amplified	2–3?	29	29	‐‐
At3g05290 (#58)	ACCGTCTTCACTATCTGAGCATC	TGGAACCAAGAACTTGCAATC	Peroxisomal adenine nucleotide carrier 1	1224	Med	‐‐	4	Amplified	4	42	42	33
At1g57770 (#59)	TTAGCGACAATGGCACCACT	AGTTCACTGGCTCCTTTATCA	FAD/NAD(P)‐binding o.r. family protein	1273	Med	2 (2)	4	Not accepted	‐‐			
At2g38040 (#60)	AATTGTGGGCAATGGCTTCG	TGGCTAATGAAACCGGTCTGG	Acetyl Co‐enzyme A carb. carb.t. α subunit?	1219	High	1 (2)	3	Accepted	‐‐			
At2g40690 (#22A)	TGCAATCACAGCTCCAGCAG	AGTTGGCAAATGCAGTCCAG	NAD‐dependent G3P dehydrogenase f. p.	1522	High	4 (2–3)	4	Not accepted	‐‐			
At5g43810 (#61)	ACATTGTGAGTGGACGCATT	TTGTTGCGTCTCAAGACTGG	Argonaute/Dicer protein, PAZ	1801	Low	‐‐	4	Accepted	‐‐			
At1g18170	No primer design possible	n.a.	n.a.	n.a.	n.a.	n.a.	n.a.	n.a.				
At3g18080 (#62)	ACTGATTGTTTAAGTGCAGCCAAAT	AACCTTAACTTCGATGCTTATCG	B‐S glucosidase 44	2275	Med	4 (2)	4	Not accepted*	32*	‐‐	‐‐	‐‐
At4g37900 (#63)	GCTGCTGTTGACAGAAACCG	TCGTGTGTATGCTTTTCTAAATGT	Hypothetical protein	2229	Med	‐‐	4	Amplified	3	34	34	34
At2g35330 (#64)	AGCCAATAGCCTCTTGAGATAC	GCTTGTTGCTTTAGGTTACGATC	RING/U‐box superfamily protein	1258	Low	‐‐	4	Accepted	‐‐			

*Note*: From the 53 loci, four (*At3g04340*, *At3g25400*, *At2g01660*, *At5g03300*) excluded based on too high best *K*, and 18 loci (*At5g66290*, *At1g57760*, *At1g49430*, *At2g25310*, *At2g40490*, *At5g66080*, *At2g28900*, *At2g45640*, *At4g32175*, *At5g61640*, *At5g63310*, *At5g17330*, *At4g33865*, *At1g55910*, *At1g60990*, *At5g11950*, *At2g04350*, *At3g15610*) rejected after visual inspection of dendrograms/alignments. Detailed information here is given only for tested loci. Locus names according to Mandel et al. (2014) plus short numbers for easier handling. Corresponding genes as annotated in the *Artemisia* genome are given. Aligned length and estimated variability (“estim. variab.”) referable to cluster consensus sequence alignments used for primer design, cut to marker length. “screen: ind. mult. bands”: number of individuals with multiple bands in the PCR screening, alongside number of PCR bands in brackets. “status after PCR screening”: the category of each marker as defined in the text; markers #50 and #62 (marked by asterisks) were amplified for comparison purposes. For the Mann–Whitney *U* test, accepted and amplified markers were coded as “yes”, all other markers as “no”. Marker #51_2 was not included into calculations. “amplif.: ind. mult. bands”: number of individuals with multiple bands during amplification of all 42 accessions; bold type and asterisks denote those where multiple bands are likely due to paralogy, underlined numbers are loci eventually producing at least one secondary contig in any individual during de novo assembly. Columns “no. seq. approach A–C/concat. ML” denote the number of sequences included in the gene trees used for the species tree and concatenation (maximum likelihood) approaches (split loci given separately).

Abbreviations: carb., carboxylase; carb.t., carboxyltransferase; d.‐c. p., domain‐containing protein; f. p., family protein; G3P, glycerol‐3‐phosphate; o.r., oxidoreductase; t. f., transporter family.

#### 2.4.2 Amplification, library preparation and sequencing

All selected loci were amplified in all 40 accessions of *Leucanthemum* and two outgroup genera. Nanopore library prep and barcoding were performed using the Native Barcoding Expansions 1–12 and 13–24 (EXP‐NBD104/EXP‐NBD114), the Ligation Sequencing kit (SQK‐LSK109), and the protocol for “Native barcoding genomic DNA” with some minor modifications. Two libraries of each 21 accessions were prepared; library 1 was run on the MinION Mk1B using two Nanopore FLO‐FLG001 Flongle flow cells, for library 2, one Flongle flow cell was used.

#### 2.4.3 Data pre‐processing

With amplified fragments being approx. 900–2500 bp long, reads were length‐filtered with nanofilt v.2.8.0 (De Coster et al., 2018) to a minimum length of 500 bp (to remove artifacts generated during PCR and library prep) and a maximum length of 3800 bp (to remove chimeric fragments erroneously ligated together during library prep). As ONT cautions users about the risk of a small proportion of reads (5%–7%) forming concatemers when amplicon libraries are prepared using ligation‐only chemistry, adequately dealing with possible chimeric sequences was incorporated into the workflow at several steps (Figure 1, “*Filter Chimeras! #1‐4*”; see also file “3_detailed_workflow2_ampliconSeq.pdf” at Dryad).

#### 2.4.4 Mapping and filtering

Demultiplexed reads from each individual were then mapped to all loci using minimap2 v.2.21 (Li, 2018, 2021). Conveniently, mapping references are based on the already existing locus‐wise cluster consensus sequence alignments comprising all individuals (see Chapter 2.3.5), after some processing also involving script 7_vsearch_testclusteringthresholds.py.

In a next step, the mappings were evaluated in various ways to identify possible problems with the mapping procedure. Additional mapping references were generated where appropriate and the respective mappings repeated. Used tools for these steps comprised script 8_filter_entirelyunmappedreads.sh, BLAST, the integrative genomics viewer (igv) v.2.4.15 (Robinson et al., 2011), samtools, script 9_extract_mapping_statistics.sh, the reformat.sh script from the bbtools suite v.38.87 (Bushnell, n.d., download website see References) and canu v.2.1. Also, mappings were filtered by dismissing unmapped, secondary, and supplementary mappings (samtools) and removing concatemeric reads (chimera filtering step #4) via checking for those with high numbers of soft‐clipped bases in mappings (reformat.sh from bbtools). For further information on the whole mapping evaluation and filtering process refer to the “detailed methods” document at Dryad.

#### 2.4.5 Read extraction and de novo assembly

From the filtered mappings, the reads were extracted with samtools fastq v.1.13 (Danecek et al., 2021) and assembled de novo for each individual and locus using canu (for parameter settings see Dryad file “4_workflow_commands.docx”). Statistics for all canu runs were generated with the script 10_extract_canu_statistics.sh. Problematic assemblies were examined manually and, where appropriate, repeated with modified settings.

### 2.5 Marker alignments and inference of phylogenetic trees

After assembly, the resulting contig FASTA files were further processed (script 11_sequencename_from_filename.sh), and then concatenated locus‐wise and aligned with mafft v.7.490. Alignments were corrected manually using bioedit v.7.2.5 (Hall, 1999) and trimmed to the length of their reference (without primer regions). Gene trees were calculated for all markers using iq‐tree v.1.6.12 (Hoang et al., 2018; Kalyaanamoorthy et al., 2017; Nguyen et al., 2015). Species trees were computed with astral v.5.7.8 (Rabiee et al., 2019; Sayyari & Mirarab, 2016; Zhang et al., 2018) using the gene trees from iq‐tree and setting the outgroup to *Chlamydophora*.

Three approaches were tested regarding the tree input for species tree generation. First, gene trees computed from unmodified markers were used as input (“unmodified” approach A). In the other two approaches, efforts were made to diminish the effects of possible paralogy still present in the chosen markers, based on strategies outlined in Yang & Smith (2014). Briefly, in the second approach, markers featuring at least one accession with >1 contig (here referred to as “secondary contigs”) were inspected for exceptionally long branches and divided into two subsets of accessions where necessary (“split paralogs” approach B). In the third approach, ALL markers from the second approach B were closely examined for possible evidence of remaining paralogy, by the help of gene trees, as well as NeighborNet splits graphs (Bryant & Moulton, 2004). Markers with exceptionally long branches were again divided into subsets. Also, where necessary, presumably paralogous elements of the trees were removed using the Rooted ingroups (RT) strategy as described by Yang & Smith (2014). The result of this process are “cleaned” subtrees with only one contig per accession; the “cleaned” gene trees were input into a third round of species tree reconstruction (“Yang and Smith” approach C). For details on the RT strategy as well as approaches A‐C refer to the “detailed methods” document at Dryad. For the species tree from the “Yang and Smith” approach C, conflicts among the underlying gene trees were examined using phyparts v.0.0.1 (Smith et al., 2015) with option *‐a 1* (thorough conflict analysis) set. Visualization of the results on the species tree was done using phypartspiecharts (Johnson, n.d., download website see References).

In addition to the quartet‐based summary method as implemented in astral, a concatenation‐based (maximum likelihood, ML) approach was tested as well, again employing IQ‐TREE. The “cleaned” datasets as created for the “Yang and Smith” approach C were used in order to avoid improper correlation of secondary contigs with identical names across different datasets. Marker alignments were concatenated into one single FASTA file and input into IQ‐TREE using the same settings as for gene tree calculations. In the resulting tree, nodes with bootstrap support <50% were collapsed using TreeGraph v.2.15.0 (Stöver & Müller, 2010).

## 3 RESULTS

### 3.1 Target enrichment data analysis

Sequencing of the enriched pool of four representative species resulted in 1,235,268 passed reads having an average read length of 2479 bp and an average read quality of 14. Statistics of the target enrichment data are available in Table 4. From the filtered reads, app. 34% were found to be on‐target according to the local *megablast* search, that is, pertaining to one of the CompCOS ESTs. Testing for the most sensible threshold for clustering reads (Table 5) provided evidence for a CT around 0.90 constituting a turning point, from whereon cluster numbers steeply increase while cluster maximum sizes drop. On the other hand, consistently more ESTs were found using higher CTs; however, obtaining a few percent more ESTs is unlikely to outweigh the considerable difficulties analyzing a triple amount of clusters. Considering that the CT should also reflect the raw error rate of Nanopore reads (app. 12% with the used chemistry), above which clusters might be separated based on mere artifacts, the CT was chosen at 0.88. Results from the clustering procedure for all four individuals are given in Table 6. All cluster centroids could be assigned to a CompCOS EST.

**TABLE 4:** Read statistics as obtained after demultiplexing of data from the target enrichment experiment with four individuals.

Sample	*L. vulgare* (120‐2)	*L. monspeliense* (131‐1)	*L. gaudinii* (276‐1)	*L. rotundifolium* (495‐2)	n.a.
Barcode	BC 02	BC 04	BC 05	BC 06	Unclassified
Obtained reads	194,073	530,407	180,222	107,008	223,541
Mean read length	1960	1791	1790	2164	4267
Mean read quality	14.3	14.3	14.3	14.3	13.7
Read length N50	2096	1902	1915	2313	4458
Total bases	380,307,721	949,968,960	322,610,173	231,595,129	953,939,974
Reads after trimming/filtering	189,959	520,131	176,041	104,969	n.a.
% bases lost	1.2	1.3	1.3	1.1	n.a.
Mean length‐filtered reads	1978	1803	1808	2183	n.a.
Reads with BLAST hit	63,581	175,120	59,012	36,258	n.a.
% reads on‐target	33.5	33.7	33.5	34.5	n.a.
Number of found ESTs	1219	1380	1255	1161	n.a.
% of all 2597 ESTs	46.9	53.1	48.3	44.7	n.a.

*Note*: Number of obtained bases and reads alongside length and quality information is referable to passed reads, percentages of reads on‐target refer to filtered reads.

Abbreviation: ESTs, Expressed Sequence Tags.

**TABLE 5:** Tests of different clustering thresholds (CT) for VSEARCH, based on on‐target reads from *L. monspeliense*.

Clustering threshold	0.80	0.85	0.88	0.90	0.95	0.97
Number of clusters	7737	14,512	24,188	36,678	126,362	169,462
Max. size of clusters [no. of reads]	706	760	659	565	145	73
Mean size of clusters [no. of reads]	23	12	7	5	1.4	1.0
% singleton clusters	53.3	68.3	75.6	79.8	91.7	98.3
% large clusters (≥100 reads)	6.6	3.1	1.5	0.7	0.007	0
Avg. % identity of reads within clusters	90.3	91.4	92.2	93.0	95.7	97.4
% of cluster consensuses with BLAST hit	99.1	99.5	99.8	99.8	99.9	‐
Number of found ESTs, consensus‐based (% of total ESTs)	1202 (46.3%)	1249 (48.1%)	1279 (49.2%)	1311 (50.5%)	1368 (52.7%)	‐
% of cluster centroids with BLAST hit	100.0	100.0	100.0	100.0	100.0	‐
Number of found ESTs, centroid‐based (% of total ESTs)	1225 (47.2%)	1270 (48.9%)	1297 (49.9%)	1321 (50.9%)	1369 (52.7%)	‐

*Note*: Obtained cluster consensuses and centroids were BLASTed against the collection of 2597 CompCOS ESTs, the number of found ESTs is given. No BLAST search was conducted for the clusters obtained with CT = 0.97.

**TABLE 6:** Results of VSEARCH clustering with clustering threshold CT = 0.88 in four individuals.

Sample	*L. vulgare*	*L. monspeliense*	*L. gaudinii*	*L. rotundifolium*
Barcode	BC02	BC04	BC05	BC06
Reads on‐target	63,581	175,120	59,012	36,258
Total basepairs	129,598,999	325,038,247	109,864,997	81,463,256
Number of clusters	10,516	24,188	9948	6453
Max. size of clusters [no. of reads]	267	659	296	194
Mean size of clusters [no. of reads]	6	7	6	6
% singleton clusters	66.5	75.6	65.2	63.1
% large clusters (≥100 reads)	0.4	1.5	0.4	0.2
Avg. % identity of reads within clusters	92.2	92.2	92.2	92.3
% of cluster consensuses with BLAST hit	99.5	99.8	99.7	99.6
Number of found ESTs, consensus‐based (% of total ESTs)	1130 (43.5%)	1279 (49.2%)	1151 (44.3%)	1048 (40.4%)
% of cluster centroids with BLAST hit	100.0	100.0	100.0	100.0
Number of found ESTs, centroid‐based (% of total ESTs)	1157 (44.6%)	1297 (49.9%)	1166 (44.9%)	1063 (40.9%)

*Note*: Obtained cluster consensuses and centroids were BLASTed against the collection of 2597 CompCOS ESTs, the number of found ESTs is given.

### 3.2 Loci summary statistics and pre‐choice loci

According to loci summary statistics (condensed information in Table 7; full statistics available at Dryad), 798 of the 1061 loci targeted by the CompCOS probe set were captured with at least one read (“enriched loci”). Across all individuals, only 52 clusters out of 51,105 had to be removed due to contradictory locus assignments in cluster centroids versus vsearch consensuses; 188 clusters could be assigned based on centroids only. In general, read numbers were strongly positively correlated across individuals; for example, in a locus where *L. monspeliense* had many reads, the other individuals were likely to have many reads as well. From the 798 loci, 323 were excluded based on too many or too few reads, another two loci did not have reads for at least three individuals, and six loci failed to have a minimum of one cluster with at least five reads in at least three individuals; this resulted in 467 pre‐choice loci for further testing. Among pre‐choice loci, averaged across all loci and individuals, 77% were low‐coverage clusters (1–4 reads; variation 42%–93% for a single locus); only 16% of the clusters had 10 reads or more (4%–50%).

**TABLE 7:** Statistics of enriched, pre‐choice and proposed candidate loci in all four individuals used for target enrichment.

	Enriched loci	Pre‐choice loci	Candidate loci
Number of loci	798	467	53
Reads per locus			
Min	1	100	104
Max	6166	999	840
Avg.	417	378	301
Clusters per locus			
Min	1	12	12
Max	864	172	132
Avg.	64	58	40
Clusters with 1–4 reads Avg. (min‐max)	‐‐	77% (42%–93%)	76% (42%–89%)
Clusters with 1–9 reads Avg. (min‐max)	‐‐	84% (50%–96%)	82% (50%–90%)
Index 1 (mean standardized reads per cluster)			
Min	‐‐	−0.9201	0.1640
Max	‐‐	2.5560	2.5560
Avg.	‐‐	0.2218	0.6883
Index 2 (*k*‐mer‐based similarity, MEAN value per locus)			
Min	‐‐	−100.00	−100.00
Max	‐‐	44.94	−32.94
Avg.	‐‐	−32.54	−71.57
Index 2 (*k*‐mer‐based similarity, STDEV per locus)			
Min	‐‐	0.00	0.00
Max	‐‐	154.98	47.94
Avg.	‐‐	49.09	28.73
Index 3 (mean entropy)			
Min	‐‐	0.0000	0.0000
Max	‐‐	3.3299	0.5305
Avg.	‐‐	0.6145	0.1262
Index 4 (silhouette coefficient with best *K*)			
Min	‐‐	2	2
Max	‐‐	28	6
Avg.	‐‐	3.29	2.62

*Note*: Minimum and maximum values across all loci are given alongside average values. “Clusters with 1‐4 reads” are low‐coverage clusters as defined in the text. For index 2 (*k*‐mer‐based similarity, KBS), statistics are given separately for its mean value and standard deviation. “‐‐” indicates that the respective value was not obtained.

### 3.3 Choice of putatively non‐paralogous loci and assessment of candidate loci

**FIGURE 3:**
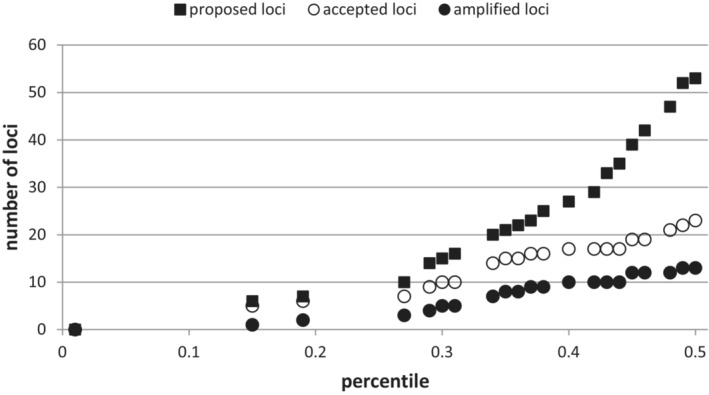
Proposed, accepted and amplified loci (see Chapters 3.3 and 3.4) at varying percentile thresholds of each index 1 (mean standardized number of reads per cluster), index 2 (*k*‐mer‐based similarity, KBS, mean and standard deviation) and index 3 (mean entropy). Squares denote the number of loci that satisfied the percentile threshold in all four index values (e.g., at the 15% percentile on the *x*‐axis, to be proposed, a locus needed to have indices scoring among the best 15% of values; here, true for six loci). Only percentiles with a corresponding increase in proposed loci are shown on the *x*‐axis.

Minimum, maximum, and average values for indices 1–4 in the pre‐choice loci are reported in Table 7. At a proposal threshold of 15% (loci among best 15% regarding values in all indices), six loci were proposed by script 5_best_loci.r; at lower values, no locus passed the filter (Figure 3, “proposed loci”; detailed account on loci proposed at different thresholds is available at Dryad). Only four more loci were added until 27% proposal threshold, while at higher thresholds the number of proposed loci steadily increased. To obtain a reasonable number of loci for testing, we chose the proposal threshold at 50%, where 53 loci were proposed (“candidate loci”). These candidate single−/low‐copy loci were characterized by considerably better index values compared to the pre‐choice loci (Table 7). Sixty percent of the candidate loci had a mean entropy of 0 (index 3). Regarding index 4, only four loci had a best *K* = 5 or *K* = 6 and consequently were excluded. Table 3 lists all proposed candidate loci alongside additional information. From the remaining 49 candidate loci, 18 were rejected after visual inspection of dendrograms and alignments of cluster consensus sequences.

### 3.4 PCR screening, accepted and amplified loci, amplification results

For three loci, primers could not be designed, the remainder was tested in the PCR screening (“tested loci”). Of these, 23 loci were regarded as acceptable in principle (“accepted loci”, see Table 3). For the purpose of the present study, five more loci were dismissed for failing to amplify in one or two individuals, and another five for having too low variability. Thus, a total of 13 loci was available for amplicon sequencing (“amplified loci”). A graphical representation of the amount of proposed, accepted, and amplified loci as present at different proposal thresholds is shown in Figure 3. It is apparent that the steep increase in the amount of proposed loci from the ca. 40% percentile threshold on is not followed by the accepted loci. On average, compared to non‐accepted loci, accepted loci were characterized by lower (=better) values for index 2 and 3 (Table 8). For index 1 (mean standardized reads per cluster), there was almost no difference. In the Mann–Whitney *U* test, after Bonferroni correction of the obtained *p*‐values and at a significance level of .05, only the entropy score showed a significant shift (p* = .0066).

**TABLE 8:** Average index values of accepted and non‐accepted loci within the 53 proposed candidate loci, and results of corresponding one‐sided Mann–Whitney *U* tests, with obtained *p*‐values given without (“uncorrected”) and with (“corrected”) Bonferroni correction.

	Index 1	Index 2, mean	Index 2, stdev	Index 3
Avg. accepted loci	0.6831	−75.1190	23.8212	0.0477
Avg. non‐accepted loci	0.6922	−68.8570	32.4873	0.1864
Mann–Whitney *U*, uncorrected *p*	0.1532	0.1195	0.0327	0.0017
Mann–Whitney *U*, corrected *p*	0.6128	0.4780	0.1308	0.0066

*Note*: Significant results (at a .05 significance level) are highlighted in red. Index 1: mean standardized number of reads per cluster, index 2: *k*‐mer‐based similarity (KBS) index, index 3: mean entropy. For index 1, higher values are better, for indices 2 and 3, lower values are better.

Apart from the 13 loci selected by the pipeline, one low‐variability locus (*At1g61040*, #50) and one locus with clear double bands in all four individuals (*At3g18080*, #62) were also included into amplicon sequencing. This was done to compare the performance of the selected loci to more conserved and likely paralogous loci, respectively. Of the 630 PCR reactions performed during amplification of the 42 individuals, 34 failed, with the respective marker‐individual combinations not being included into library prep and sequencing. Of the 15 markers, the amplicons of only four had single bands in all of the amplified individuals; four markers (including #62 as expected, plus #45, #9A, and #54) had several individuals with amplified multiple bands, the latter mostly showing constant, characteristic fragment lengths across several species, which might point towards hidden paralogy in the respective markers (Table 3; detailed information on PCR bands of individual accessions is available in the canu statistics table at Dryad).

### 3.5 Amplicon sequencing data analysis

The two runs of library 1 (21 individuals comprising 11 species) yielded 573,382 passed reads (894 megabasepairs, Mbp), library 2 (21 individuals comprising 10 species plus the two outgroups) yielded 309,167 passed reads (483 Mbp). The mean read quality was 11.8/11.6 and 11.8, respectively. 2.2% and 2.9% of reads were excluded due to chimeric or supplementary mapping (“filtering of chimeras #2”). Demultiplexing yielded between 9158 and 29,784 reads per individual.

The VSEARCH clustering used to minimize the number of mapping references per locus resulted in a considerable reduction of references: 11 of 15 loci had only one single reference afterward. Marker #45, #54, and #58 each had two references (the divergent sequences splitting from the others at CT = 0.65, 0.65, and 0.70, respectively), marker #51_1 had three references (CT = 0.65 and 0.73). Reference FASTA files for all loci are available at Dryad. Between 10.0% (in *L. gaudinii* and *L. vulgare*) and 34.6% of the reads (in *L. legraeanum*) remained entirely unmapped; for the outgroup, these proportions were 18.2% in *Rhodanthemum* and 36.5% in the more distant *Chlamydophora*. Only few of the unmapped reads BLASTed successfully against the marker references (0.06%–1.93%), with the exception of *Chlamydophora*, where 2.94% of reads had a hit, mainly to marker #54. Consequently, an outgroup‐specific reference was created and used for repeating the mapping for *Chlamydophora* with marker #54 (see 2.4.4).

Of the 596 mappings, 26 mappings had <100 reads mapped (all mapping details, including those after filtering steps, are available at Dryad). Four of these could be processed further (two with the help of an additional *L. rotundifolium*‐specific reference for marker #62 assembled in canu), the others were excluded. Many cases concerned mappings with very low read numbers in marker #13A and #46; the cause for this remained unknown. Further detection of poor mappings via the number of soft‐clipped bases (threshold >50 bp) revealed 30 problematic cases. The vast majority of those was confined to marker #62, which necessitated a third reference (based on *L. eliasii*); the mapping was repeated for all individuals with all references. The filtering of reads with soft‐clipped bases at a higher threshold (>300 bp) resulted in a loss of only 2.1% of all reads.

### 3.6 De novo assembly using canu

In four out of 569 assemblies, no contig was assembled (all canu assembly details available at Dryad); the assemblies were repeated with modified settings (increased *readSamplingCoverage* parameter or estimated genome size, and/or setting *corMinCoverage* to zero), whereby contigs were obtained. From five assemblies with <120 input reads executed manually with adapted settings, only one failed (*Rhodanthemum* for marker #56). Twenty‐four problematic assemblies were recognized and checked visually. Altogether, 66 secondary contigs were assembled, mostly in markers #45, #54, and #62, three markers with suspected hidden paralogy (see 3.4). Due to unknown reasons, not a single secondary contig was assembled in marker #9A, despite 17 individuals with two or even three bands in the PCR indicating possible shorter as well as longer copies. Generally, most problems were found in marker #62 (and to a lesser extent also marker #56). All assemblies of marker #62 were thus repeated with an increased estimated genome size to account for expected paralogs, plus lowered *minReadLength*/*minOverlapLength* to include shorter marker variants. Altogether, only one out of 574 assemblies failed after all optimizations.

### 3.7 Gene tree/species tree reconstruction

**FIGURE 4:**
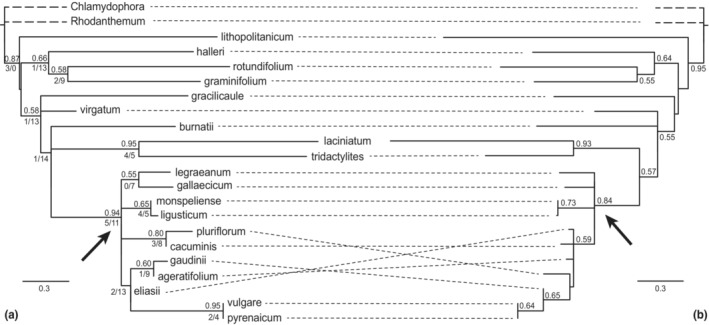
Species trees for the genus *Leucanthemum* inferred with astral, based on IQ‐TREE gene trees from (a): 15 locus alignments from the “Yang and Smith” approach C, and (b): 14 locus alignments from the “unmodified” approach A. *Chlamydophora tridentata* was set as the outgroup. Local posterior probabilities above .5 are given above branches. Values below branches are results from the phyparts analysis and denote the numbers of gene trees, which, at the respective node, are concordant with the species tree or with a conflicting bipartition, respectively. In some cases, the same gene tree can support both the species tree and conflicting topologies. Branch lengths are in coalescent units, branches of *Chlamydophora* and *Rhodanthemum* are shortened and not to scale. The clade representing the *L. vulgare*‐group is indicated by arrows.

Of the 13 amplified markers plus markers #50 and #62, six had at least one secondary contig assembled in an individual (i.e., markers #45, #51_1, #54, #57, #58, and #62; see canu statistics table at Dryad). Species tree reconstructions were done using the 13 markers yielded by the pipeline, plus marker #50 to add more information to the dataset; the paralog marker #62 was not included. All marker alignments as used for approaches A–C, for NeighborNet calculations and as basis for the concatenated matrix, also marker #62, are also available at Dryad, as are the gene trees from the unmodified markers. The astral species tree based on these markers (“unmodified” approach A) is shown in Figure 4b. Regarding the “split paralogs” approach B, gene trees from markers #45 and #54 (and expectedly, although not included, #62) showed a very distinct pattern of two subgroups subtended/separated by one or two long branches, representing a clear sign of paralogy. All three markers were split into two. In #51_1, three individuals were subtended by a single long branch and were removed. The species tree based on the resulting 16 markers including split markers #45_p1 and #45_p2, and #54_p1 and #54_p2, and the corresponding modified gene trees alongside NeighborNet splits graphs are available at Dryad. In the next step, these gene trees and splits graphs were closely examined for signs of remaining paralogy and leaves pruned where necessary (see Table 3), following the approach as described in the “detailed methods” document at Dryad (modified after Yang & Smith, 2014). All markers still having secondary contigs (#45_p1, #51_1, #57, and #58) were modified, plus markers #9A and #50. Marker #13A was dismissed completely due to a combination of two long branches and too few remaining leaves in subtrees. Thus, 15 loci were used for creating the species tree according to approach C (“Yang and Smith”), which is presented in Figure 4a.

The topology of this species tree reconstruction was mostly weakly supported. A group of taxa closely related to *L. vulgare* (the “*L. vulgare*‐group”, see Ott et al., 2022) received high support (local posterior probability PP: .94, see arrow). The same applied to the sister‐group relationships of each *L. laciniatum*/*L. tridactylites* and *L. vulgare*/*L. pyrenaicum* (local PP: .95). Weaker supports pertained to the ingroup as a whole (local PP: .87) and to a clade of *L. pluriflorum* and *L. cacuminis* (local PP: .80). The remainder of relationships was very weakly supported to unsupported. The species tree from the “unmodified” approach (Figure 4b) was even less supported. Analysis of possible conflict (using phyparts) among the gene trees used for species tree calculation in the “Yang and Smith” approach C revealed that at any given node, indeed only very few gene trees supported the respective bipartition. However, there was no well‐supported alternative bipartition either; instead there seemed to be many alternative bipartitions of which none was clearly supported. In addition, especially in the *L. vulgare*‐group, supports in gene trees were frequently below 50% and thus not reliable (see detailed phyparts results as available at Dryad).

The ML tree, by contrast, was unresolved or weakly supported only in its more basal parts (tree likewise available at Dryad). Thirteen of the 20 ingroup taxa were retrieved monophyletic, among those all nine taxa from the ancient grade. In the seven species whose pairs of individuals did not form sister groups (marked by asterisks in the tree), high supports were frequently found (BS = 51–99, in seven cases >BS = 75). The well‐established bipartition among the paraphyletic group of more ancient lineages and a monophyletic group of younger lineages did not exist. On the contrary, six taxa from the former group (*L. lithopolitanicum* through *L. burnatii*) formed a highly supported monophyletic clade, while there was no monophyly for taxa from the younger group.

## 4 DISCUSSION

### 4.1 Strategies for analyzing target enrichment data

Target capture combined with subsequent Nanopore sequencing is no longer an unusual approach. For example, Giolai et al. (2017) enriched a set of NB‐LRR (nucleotide binding‐site leucin‐rich repeat) genes from *Solanum americanum* via a commercial custom bait panel, and Bethune et al. (2019) created long‐range PCR‐derived baits for capture of long‐fragment plastome sequences with subsequent de novo assembly of chloroplast genomes. However, there still is a lack of standardized pipelines to analyze long‐read target capture data (Andermann et al., 2020). The central step in processing the data is the assembly of reads as well as the assignment to their respective gene as represented in the probe set. For Illumina data, this can be automated using, for example, the hybpiper pipeline (Johnson et al., 2016), which maps or BLASTs reads to target loci first, then performs de novo assembly of mapped reads and finally extracts exons and/or introns from the assembled contigs. hybphylomaker (Fér & Schmickl, 2018) by contrast maps reads to a pseudo‐reference previously generated from concatenated probe sequences, and consensus calling based on mapped reads is performed; the obtained consensus is then fragmented into its exonic parts.

The two main strategies for read assignment employed by these two pipelines (1: mapping, 2: BLASTing reads) have also been applied independently, mainly using Illumina but also Nanopore data. (1) Nanopore target reads were mapped against a reference, for example, by Eckert et al. (2016), but this strategy is not possible for the present study due to the lack of a genomic reference for *Leucanthemum*. Alternatively, for a set of probe loci, a pseudo‐reference as in hybphylomaker might be used; however, the larger and less standardized read length of Nanopore reads renders this a quite unfunctional task. (2) BLASTing reads against probe sequences is frequently employed for the identification of on‐target Illumina and Nanopore reads (Giolai et al., 2017; Mandel et al., 2014) and does not necessitate any other references for read assignment; thus, we chose to follow this strategy. To extract reads of interest while enabling assessment of intra‐locus variability and separation of putative paralogous sequences as far as possible, we employed a sequential clustering strategy, first extracting reads belonging to the CompCOS loci via BLAST (adapting settings by Giolai et al., 2017) and then clustering these on‐target reads with VSEARCH; assignment of each cluster to a CompCOS locus was subsequently done via another BLAST search. In contrast to de novo assembly as performed by hybpiper, we then built lamassemble consensuses from the aligned reads of each cluster to obtain representative sequences (see below), similar to the approach in hybphylomaker.

### 4.2 Clustering and cluster consensuses

Using clustering as means of capturing locus diversity is not completely free of issues. Ideally, each single‐copy locus is expected to yield one or two clusters in a diploid. However, many more clusters were usually found for loci as well as ESTs, for example, an average of eight (in *L. rotundifolium*) to 30 (in *L. monspeliense*) clusters per locus per individual; the amount of clusters seemed to be positively correlated to total read numbers. The reasons for this are apparent upon inspecting read/cluster consensus alignments of a locus. Two of those are available, for a “very good” and a “very bad” locus (*At3g05230* and *At1g01050* as presented in Figure 2), at Dryad due to their large size. Cluster consensuses are aligned alongside reads of singleton and low‐coverage clusters, which were excluded from most computations. The gappy, “perforated” alignment even in exonic regions (marked by the three EST sequences) of the “good” marker *At3g05230* shows that much of the variability found among reads and also cluster consensuses is caused by Nanopore sequencing errors, which thus have the greatest influence on the cluster generation process. Due to the chosen high threshold (which is necessary to avoid lumping closely related paralogs into the same cluster), singleton clusters are expected to be numerous, which was also observed here. The occasionally bad quality of read beginnings and ends might raise their number even further, but this problem could be alleviated by more rigorous character‐based quality trimming. Systematic Nanopore errors (e.g., from homopolymers) are expected to result in low‐coverage clusters, which are, however, easily filtered (see below). Artificial cluster splitting due to non‐overlapping cluster subregions/reads was not observed and seems to be a minor problem, but cannot be ruled out entirely; splitting might happen if a locus lacks long enough reads that act as conjunctive elements in the clustering of shorter reads. Altogether, the high degree of baseline variability (i.e., background noise) found even in the “good” locus is mainly caused by errors and artifacts and does not contain any biological meaning. Newer ONT chemistries that have become available recently and provide greater raw read accuracy (Q20+ and higher) will help to largely remove this background noise.

In the “bad”, that is, presumably paralogous locus *At1g01050*, the same effects are present; however, a much greater variability even among consensuses/reads of the same individual is observed. The traces of presumable paralogs can be seen in (consensus) sequences which are barely alignable outside the exonic regions (due to sequence divergence) and/or the respective gene (due to paralogs being located next to different neighboring genes). In *At1g01050*, three different sequence variants, each represented by at least two consensuses, can be identified outside and within exonic regions and in consensuses as well as raw reads. These variants are traceable even through the disturbing influence cast by the sequencing errors and correspond to the three main clades of locus *At1g01050* in Figure 2a. It seems very clear that this strong signal is responsible for the very bad evaluation of the locus by the pipeline.

To simplify data handling and to attenuate the negative effects of Nanopore raw read errors, for calculation of indices 2–4, singleton and low‐coverage clusters (with less than five reads) were removed as likely representing artifacts. As the quality of a consensus decreases with the number of reads it represents, setting the minimum reads per cluster to a value >5 would be desirable; however, the trade‐off between removing errors but no true signal from the dataset always has to be considered. Non‐filtered clusters were reduced to their consensus sequences, thus eliminating a considerable portion of random sequencing errors. The concept thereby is to consolidate the total variability found in reads of a locus (regardless of its causes) into few representative sequences, which can then be searched for signs of paralogy via indices 2–4.

### 4.3 Choice of single−/low‐copy loci via specialized indices

The pre‐selection procedure of loci prior to index calculation was carried out considering mainly pragmatic reasons. However, dismissal of loci with too few (<100) reads resulted in exclusion of 30% of enriched loci from any further consideration. As single‐copy loci are expected to be represented by a low number of reads a priori, it is possible that suitable loci were discarded by setting this threshold. This problem could be alleviated by increasing the total number of reads sequenced, for example by aiming at 400,000 passed reads per individual.

Altogether, the pipeline performed well in predicting low‐copy loci in *Leucanthemum*. Almost all loci proposed at low proposal thresholds (up to the max. 27% percentile) turned out to be acceptable for use (Figure 3). Limiting manual examination of loci to those included in the 30% proposal threshold instead of the chosen 50% would have yielded a proportion of about two third acceptable loci. A short evaluation of the performance of each index is provided below; generally, for all indices, better values were observed in the “candidate loci” relative to the “pre‐choice loci” (see Table 7 and file “6_full_locus_statistics.xlsx” at Dryad).

#### 4.3.1 Index 1 (mean standardized number of reads per cluster)

Results from this index were somewhat equivocal: there was almost no difference in values between accepted versus non‐accepted markers (Table 8). A possible problem is that the index generally penalizes loci with a small total number of reads. This will disadvantage single‐copy loci characterized by few reads per se. Indeed, very bad values (<−1) for index 1 are exclusively assigned to enriched loci with 1–104 reads in the present dataset. Loci with few reads can also score very well; on the other hand, however, loci with a larger number of reads will never score too bad. Also, the explanatory power of the index can be affected in cases where the number of clusters is increased artificially, for example due to Nanopore errors or a random lack of long reads in a locus/EST. These facts might explain the somewhat limited utility of the index.

#### 4.3.2 Index 2 and 3 (*k*‐mer‐based similarity, KBS, and mean entropy)

Both indices perform well in predicting low‐copy loci from the *Leucanthemum* dataset, especially the mean entropy, where the difference among values in accepted versus non‐accepted loci (within the 53 loci proposed by script 5_best_loci.r) is highly significant (Table 8). Both indices, however, suffer from the fact that individuals with only one consensus always will be scored best (−100 and 0, respectively, see Figure 2c).

Index 3 follows the principle of first clustering and then verifying whether the most relevant clusters (determined by the best value of *K*, see below) are compatible with the species affiliation of the leaves in the tree. This means that naturally, the mean entropy depends on the underlying best *K*. On the other hand, depending on the best *K*, this index is better able to tolerate situations where not all species are forming exclusive clusters, a realistic scenario in a single locus of possibly limited informative value. In marker *At1g01050* (Figure 2a), the high mean entropy of 1.3610 well reflects the consensuses of especially BC04 and BC05 emerging all over the dendrogram.

The KBS index (index 2) on the other hand is independent of clades in a dendrogram and associated assumptions, as it is based on distances only. In contrast to index 3, its principle works the other way around: the starting point are the species, and it assesses how far their sequences tend to cluster together, and how consistently so, across all included taxa. Using this approach, effects of recent duplications affecting only few taxa might become visible in the standard deviation of the species' mean KBS indices, and can thus be accounted for. A disadvantage is that two short sequences, which belong to the same paralog but cover different parts of its sequence, will artificially deteriorate the KBS index. Index 3 is less affected by this problem due to being based on dendrograms calculated using single‐linkage clustering. A possible solution to this problem in the KBS index would be to revert it to the concept it was originally inspired by, that is, the LB score (long‐branch score) presented by Struck (2014). This score is similar to the KBS index, except for the fact that it uses patristic distances for calculation and thus, similar to index 3, is based on an underlying tree. Originally designed to detect long‐branch attraction within a phylogenetic tree, the LB score is defined as the mean pairwise patristic distance of a taxon to all other taxa in the tree, relative to the average pairwise patristic distance across all taxa. It is, however, used on trees with one single accession per taxon and calculated for each leaf of the tree, so is not easily applicable here. Anyway, results for index 2 in its present shape already seem to match nicely the conditions as seen in the respective dendrograms of the loci (compare Figure 2): in marker #52, the sequences of all individuals cluster quite well but are arranged in grades in two of four individuals. The corresponding KBS is slightly increased compared to the “perfect” marker #41, in both mean and standard deviation.

#### 4.3.3 Index 4 (silhouette coefficient)

An inherent problem with this index is that the silhouette coefficient is defined for ≥2 clusters only. Consequently, a perfectly homologous locus can never receive a best *K* value = 1 (see *K* = 2 in Figure 2c). Moreover, if there is perceptible internal sub‐structure (due to general phylogenetic variability of the individuals chosen for target capture) within the locus, it might be assigned an artificially high value of *K*. Despite these limitations, the index performed quite well in the present study as a basis for the entropy index (see above), and as an additional criterion for excluding loci. The calculated index does often, but not always, correspond to the decision a human observer would have made based on the dendrogram (see Figure 2a,b).

Altogether, the combination of good index values results in the very low proposal threshold of marker #41 (Figure 2c), which is among the very first loci proposed by the pipeline. Marker #52, due to its somewhat inferior index values, was only proposed at the 34% percentile, however, performed extraordinarily well during amplification, and likely is not affected by (visible) paralogy in *Leucanthemum*. This would argue for extending the proposal threshold beyond 30% when investigating proposed loci. Marker *At1g01050*, which scored rather badly in general and very badly for index 3, is proposed only at the 94% threshold and is certainly not to be considered in studies relying on single‐/low‐copy loci.

### 4.4 Amplicon sequencing

Several tools for automatic handling of Nanopore amplicon data are available. Examples are nanortax (however limited to 16S rRNA amplicons only and intended for taxonomic analysis of microbial community samples, Rodriguez‐Perez et al., 2021) or decona (for processing data from demultiplexing to consensus calculation, Doorenspleet et al., 2021). However, none of these tools can adequately handle paralogs present in amplified loci. Thus, amplicon data here were processed manually, by first mapping the reads to references of the amplified loci and then de novo assembling reads of each locus and individual separately.

To obtain suitable mapping references from the target capture data and thus ensure effective mapping of divergent taxa, a major part of a group's variability should be (preferably evenly) represented in the individuals used for target enrichment. An alternative to de novo assembly of mapped reads is variant calling from the mapping and subsequent creation of a modified reference based on the called variants. While this can represent a suitable alternative to de novo assembly in several cases (Scheunert et al., 2020), it was found to be inappropriate in the present study (results not shown). Some tools (e.g., bcftools mpileup; Li et al., 2009) require very close references for producing correct results, a condition that cannot always be met. Furthermore, generally, only a single consensus sequence per mapping file will be produced. This means that paralog signal in the reads is either filtered completely or combined into a mixed, artificial sequence.

### 4.5 Inferring paralogy and dealing with paralogous loci

#### 4.5.1 Existing approaches and strategy followed in this study

A considerable amount of literature has been published dealing with the topic of orthology inference (which often goes along with the exclusion of identified paralogs). Orthology prediction methods may be tree‐based or graph‐based (see Altenhoff et al., 2019, and Fernández et al., 2019, for two reviews). A wealth of specialized databases and tools is currently available (e.g., oma gethogs, Altenhoff et al., 2013; orthofinder, Emms & Kelly, 2019; the orthodb database, Zdobnov et al., 2021), however mostly, these approaches are tailored to WGS (Whole‐Genome Sequencing) or transcriptomic Illumina datasets. A suitable solution for selection of presumably paralog‐free markers from target capture long‐read data has, therefore, been missing until now.

With the well‐known history of WGDs and polyploidizations in the Asteraceae in mind, and considering the pronounced occurrence of polyploidy in *Leucanthemum* itself (representing a “worst‐case scenario” for testing), the question was whether single‐copy markers existed in the genus at all. So, we pursued a two‐tier approach, by first removing paralogous loci as far as possible, and then detecting and removing potential paralogous elements in the chosen markers where needed. Locus exclusion in paralog‐rich groups, however, carries the risk of significantly reducing the number of available loci; in the worst case, there simply might be no single‐copy genes, which are present in all of the species studied (Emms & Kelly, 2018; Lee et al., 2013; Yan et al., 2022). This effect was also observed in *Leucanthemum*, with only 13 loci finally found suitable for amplification, and within these, the majority still carried traces of paralogy (no or almost no indications of possible paralogy only in markers #41, #46, #52, #63, and possibly #44 and #56).

#### 4.5.2 Using canu for paralog detection within amplicon data

Evidence for remaining paralogous components within loci for this study essentially came from multiple bands observed during amplification, and from secondary contigs assembled by canu. The relationships among these two factors were not always straightforward: while generally, markers with constant, characteristic double, or even multiple bands also assembled secondary contigs, this was not true for every single individual. On the other hand, in several cases, a secondary contig was assembled where there was only a single band in the PCR (see the canu statistics table at Dryad). A possible explanation for the complete lack of secondary contigs in marker #9A, given the very limited length variation of the reads in the mappings, might be that potentially divergent copies could not map to the references provided or did not bind to the capture probes in the first place.

An important issue are the assembly strategies implemented in canu: in general, larger systematic (consistent) differences in the underlying reads resulted in several contigs. There are, however, some exceptions to this rule, where no separate contigs may be assembled: (1) number of available reads is too low for a given “sequence variant” (presumed to be a paralog); (2) sequence variants merely involve a large deletion; (3) sequence variants mainly differ in repeat motives; (4) sequence variants are too similar (threshold roughly estimated at about 95%). Apparently, the performance of canu in detecting paralogs decreases as these get more similar, which means that the approach becomes increasingly unfeasible in very young lineages with recent duplication events, and that manual checking is always necessary. Morales‐Briones et al. (2022), using Illumina target capture data with hybpiper, also noted the problem of inadvertently merging paralogs with high sequence similarity into one. In plant groups prone to hybridization and with recent allopolyploidy or even homoploid hybrid speciation, another general drawback is that, apart from paralogous copies, homoeologs might also be assembled, which confounds the correct detection of remaining paralogy. Although only diploid taxa were included in the present study, an influence exerted by potential hybrid speciation and/or introgression in single taxa cannot be ruled out.

#### 4.5.3 Tree‐pruning as means of eliminating paralogy from datasets, and the issue of missing data

Apart from dismissing whole loci due to presumed paralogy, separating or pruning supposedly paralogous elements in gene trees is an alternative method of ensuring orthology in a dataset, and was adopted, for example, by Liu et al. (2019) and Karimi et al. (2020). In the present study, tree‐based pruning was used to remove remaining traces of paralogy, as per our definition detectable via secondary contigs but also non‐monophyletic outgroups. We hereby followed Yang & Smith (2014) and their RT method (see Chapter 2.5). Approaches by the latter authors were also employed by Morales‐Briones et al. (2022), however, using Illumina data.

Unfortunately, employing the RT method in the way described here resulted in a substantial increase of missing data in the markers that had to be modified, with gene trees losing an average of 27.3% of their leaves. It has been argued that species tree inference methods like ASTRAL are robust to missing data (e.g., Nute et al., 2018; Xi et al., 2016); indeed, the species tree based on gene trees from the “Yang and Smith” approach yielded a similar topology as that based on the “unmodified” approach.

#### 4.5.4 Recommendations for future use of the workflow

For future application of the pipeline, some recommendations can be given: (1) Individuals for the target capture experiment should be evenly distributed across the variability of the group. (2) Employing sufficient sequencing depth per individual will alleviate problems related to low‐ and lower‐coverage clusters and to lack of data in general, and will also provide enough reads in single‐copy loci to prevent their inadvertent exclusion. (3) If longer loci than the ones produced here are desired, the DNA shearing, size‐selection procedures, or Nanopore library prep in the target capture experiment can be modified accordingly, to eventually produce longer sequenced reads. This would also lead to both better and longer consensuses, improving calculation of indices 2 and 3 and reducing the risk of artificial cluster splitting even further. (4) The amount of loci obtained by the pipeline is to some extent adjustable by the user. In *Leucanthemum*, the proportion of acceptable loci decreases as more and more loci are proposed, but even at higher proposal thresholds, good loci can be obtained, at the price of more laborious manual examination and PCR‐testing. How to deal with this trade‐off depends on the study envisioned, and might, of course, vary in different plant groups. In principle, the approach for identifying single‐copy loci from target capture data as presented here would be also feasible for use with long reads from Pacific Biosciences (PacBio) sequencing. Some results might even be improved, for example, regarding the clustering process or the performance of index 1; this would, however, come with the disadvantage of higher sequencing costs. The analysis of such data additionally would require tools tailored to PacBio or Illumina data in some cases.

#### 4.5.5 Phylogenetic inferences

One aim of the present study has been the establishment of a suitable molecular marker set, which could be used for the reconstruction of phylogenetic relationships among the diploid representatives of *Leucanthemum*, and possibly also for disentangling the phylogenetic history of its polyploid lineages in future studies.

Species tree reconstruction based on multilocus sequence information is nowadays accomplished either by concatenation of individual marker alignments and a “total evidence” analysis with gene tree reconstruction methods (e.g., maximum likelihood, Bayesian inference), or with more sophisticated species tree reconstruction methods, for example, based on coalescent theory. It has been asserted that the concatenation approach may produce robust and well‐supported, but inaccurate phylogenetic reconstructions (Kubatko & Degnan, 2007; Weisrock et al., 2012). This observation is corroborated by the present study: while an overwhelming number of monophyletic groups receive high support in terms of bootstrap supports in the concatenation‐based maximum likelihood tree (available at Dryad), its topology (especially regarding the position of members of the ancient grade of taxa here placed within a derived, monophyletic clade, while by contrast the *L. vulgare*‐group lacks monophyly) is not only in sharp contrast to the astral species trees (Figure 4), but also to all other hitherto published phylogenetic reconstructions based on alternative marker sets, that is, nrDNA ETS (Oberprieler et al., 2014), plastid and low‐copy nuclear markers (Konowalik et al., 2015; Wagner et al., 2019), and 9248 RADseq loci (Ott et al., 2022). Therefore, the species tree reconstruction based on astral appears more trustworthy, despite the disappointingly low support values for many of its monophyletic groups. It has to be concluded that approaches based on only few markers, as the one presented here, are unsuitable for effectively reconstructing evolutionary relationships among diploid *Leucanthemum* species; this is better accomplished using RADseq data (as shown in Ott et al., 2022).

Both astral species trees in Figure 4 corroborate the well‐known bipartition of *Leucanthemum* diploids into two groups: the ancient, paraphyletic assemblage of *L. lithopolitanicum* through to *L. laciniatum* and *L. tridactylites*, and the closely knit, monophyletic group around *L. vulgare*. The results from the phyparts analysis, revealing large amounts of discordance among gene trees even in nodes supporting well‐established relationships, suggest that low supports in the species tree are indeed caused by gene tree conflict (although, especially within the *L. vulgare*‐group, a lack of phylogenetic signal also seems to play a role). This conflict may be attributable to ILS and/or gene flow across lineages (hybridization among species or homoploid hybrid speciation). Konowalik et al. (2015) proposed the latter as the main accountable factor; however, the assumed single‐copy nature of nine of the loci used in that study (previously proposed by Chapman et al., 2007) has never been verified for *Leucanthemum*; the gene tree incongruence observed may thus also have been due to artifacts caused by unrecognized paralogy.

Upon comparison with the chloroplast genome‐based gene tree in Konowalik (2015; supplemental material, figure 11, based on five intergenic spacer regions), a mostly congruent pattern regarding species relationships emerges. Many of the more ancient species in Figure 4a (from *L. lithopolitanicum* through *L. tridactylites*) are part of a monophyletic clade in the chloroplast tree, and the well‐supported monophyletic group of *L. legraeanum* through *L. pyrenaicum* is also nearly identical with a cpDNA haplotype clade. However, three exceptions to this pattern are observable, involving unexpected positions of *L. pluriflorum* plus *L. virgatum*, one sample of *L. graminifolium*, and one of *L. burnatii* in the chloroplast tree. While members of the *L. vulgare*‐group have a widespread distribution, several relictual endemics are found in the ancient grade of *Leucanthemum*. Considering also the known tendency of active evolution by hybridization events (especially connected with polyploidization, cf. Oberprieler et al., 2023), the transfer of chloroplasts out of the *L. vulgare*‐group into lineages of the more ancient *Leucanthemum* grade here appears a reasonable explanation for the cases of plastid‐nuclear tree incongruence.

The overall concordance observed among the plastid tree and the nuclear species tree in Figure 4a, however, could argue for ILS rather than hybridization as responsible factor for the nuclear gene tree incongruence, given also the short internal branches relative to long terminal ones in the paraphyletic and ancient group and overall short branches in the monophyletic, more recently diverged *L. vulgare‐*group in Figure 4a. Retention of ancestral polymorphism could have been caused by two epochs of fast speciation events (radiations) in the evolutionary history of the genus, one in an early phase with the onset of the glaciation cycles in the Early Pleistocene (Gelasian or Calabrian, 2.58–0.8 million years ago) and the other in a more recent one around 500,000 years ago (Chibanian; see chronogram in Wagner et al., 2019).

## 5 CONCLUSIONS

In the present study, we introduced a new method for mining target enrichment data from a genus of interest for single‐copy nuclear loci. The presented pipeline renders this a manageable task at comparatively low cost. Our approach is based on Nanopore read data, leveraging the power of long reads, which are essential for exact identification of paralogous copies, together with straightforward sequencing even in very small labs. We have shown that sequential clustering (BLAST plus VSEARCH) provides an elegant way of assigning captured reads to their probe sequences and to assess locus variability. The four presented indices for the extraction of suitable loci proved effective, with some limitations only for index 1 (number of reads per cluster). For detection of remaining paralogy in the chosen, amplified markers, canu de novo assemblies of mapped amplicon reads were also employed. Our results show that although canu does not perform 100% correctly in generating representative contigs for all presumed paralogous copies contained within a given marker's reads, it is a good indicator of the presence of paralogy, and its performance is likely to still increase in older lineages. Subsequent tree‐based pruning of suspected paralogous elements can result in substantial amounts of missing data in groups with generally high levels of WGD like the Asteraceae. In families not as intensely influenced by polyploidization, our pipeline can be expected to yield a larger number of suitable loci requiring less post‐sequencing modification with regard to remaining paralogy. In difficult genera like *Leucanthemum*, conflicting phylogenetic signal among the obtained markers might lead to problems in downstream analyses aiming to reconstruct diploid relationships (as seen here), and thus likely also in analyses examining allopolyploid origins. In the latter case, using only an optimal subset of the found markers (those which did not require modification after amplification and sequencing—six loci in this study) might help to enable successful analyses; as demonstrated in different studies (Freyman et al., 2023; Jones et al., 2013), very few markers can be sufficient for this purpose.

## AUTHOR CONTRIBUTIONS

**Agnes Scheunert:** Conceptualization (equal); data curation (lead); formal analysis (equal); investigation (lead); methodology (lead); visualization (lead); writing – original draft (lead); writing – review and editing (lead). **Ulrich Lautenschlager:** Formal analysis (equal); methodology (equal); software (lead); writing – review and editing (equal). **Tankred Ott:** Formal analysis (equal); methodology (equal); software (equal); writing – review and editing (equal). **Christoph Oberprieler:** Conceptualization (equal); funding acquisition (lead); methodology (equal); resources (lead); writing – review and editing (equal).

## CONFLICT OF INTEREST STATEMENT

The authors declare no conflict of interest.

## Data Availability

All base‐called, passed, demultiplexed raw reads generated in the target enrichment experiment and from amplicon sequencing are available at the NCBI Sequence Read Archive (SRA) under BioProject ID PRJNA952107. Various supplementary materials, including a detailed methods document alongside two detailed workflow figures, used BASH and/or AWK commands alongside explanations, FASTA reference sequences used for mapping of the amplicon sequencing reads, all marker alignments, and detailed statistics tables as well as phylogenetic trees and NeighborNet splits graphs not shown here are available at Dryad (doi: 10.5061/dryad.2fqz612tm). The scripts written for and used in the present study, together with explanations and usage instructions are additionally available on GitHub (at https://github.com/AGOberprieler/Nano‐Strainer). All remaining data are presented in the main body of this article.
